# Operando Transmission Electron Microscopy Insights Into the Evolution of Cu_2_O–SnO_2_‐Based Catalysts During CO_2_RR

**DOI:** 10.1002/cssc.202502705

**Published:** 2026-04-22

**Authors:** Cecilia Irene Gho, Katarzyna Bejtka, Marco Fontana, Federica Zammillo, Hilmar Guzmán, Micaela Castellino, Alberto Lopera, Mariajosé López‐Tendero, Roger Miró, Miriam Diaz de los Bernardos, Simelys Hernández, Angelica Chiodoni

**Affiliations:** ^1^ Center for Sustainable Future Technologies @Polito Istituto Italiano di Tecnologia Torino Italy; ^2^ Department of Applied Science and Technology Politecnico di Torino Torino Italy; ^3^ Laurentia Technologies Parque Tecnologico Valencia Spain; ^4^ Unitat de Tecnologia Química, Eurecat Centre Tecnològic de Catalunya Tarragona Spain

**Keywords:** CO_2_RR, copper‐based catalyst, electrochemistry, electron microscopy, operando liquid phase TEM

## Abstract

The electrochemical reduction of CO_2_ into valuable products is a promising strategy for mitigating atmospheric CO_2_ emissions, particularly coupled with solar energy. Among the possible products, CO is currently most attractive, both because it can combine with hydrogen to produce syngas, and because CO is a key reactant in the chemical industry. Copper‐based electrocatalysts are extensively investigated for CO_2_ reduction; however, their morphological and chemical evolution under operating conditions still needs to be clarified, to understand the relationship between morphology, structure, and catalytic activity. This paper discusses a Cu_2_O–SnO_2_ based‐catalyst, designed to enhance CO selectivity, and studies its evolution under reaction conditions by operando electrochemical liquid‐phase transmission electron microscopy (EC‐LPTEM). First, the morphology and composition of the as‐prepared catalyst is characterized. Then, operando EC‐LPTEM is discussed and compared to the post mortem catalyst characterization, together with electrochemical behavior evaluated in the lab‐scale device. Different experimental conditions were studied to provide insights on how the catalyst modifies during the electrochemical activity. This characterization contributes to a better understanding of the possible mechanisms involved in the CO_2_ reduction, and of the factors influencing the catalyst stability and selectivity, supporting the development of improved catalysts for CO_2_ to syngas conversion.

## Graphical abstract

Proper CO_2_ management holds a key role in the energy transition, and electrochemical CO_2_ reduction to CO plays pivotal role in this process. Cu_2_O–SnO_2_‐based electrocatalyst is studied under electrochemical operative conditions, combining lab‐scale and operando liquid‐phase electrochemical transmission electron microscopy. This approach reveals how the catalyst restructures during operation and sheds light on its stability and activity.

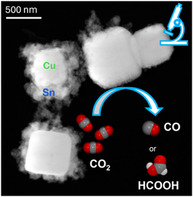

## 1 Introduction

Electrochemical conversion of CO_2_ into carbon monoxide (CO) is a crucial pathway toward carbon capture and utilization, as CO serves as a key feedstock for syngas and numerous downstream chemical processes. However, the inherent complexity of the process requires an optimized catalyst. While noble metal catalysts (e.g., Ag, Au) are highly selective toward CO, their excessive cost and scarcity motivate research into earth‐abundant, noble‐free alternatives. Copper–tin (Cu–Sn) catalysts have emerged in last years as a promising class in this respect, owing to the synergistic effects between Cu and Sn that allow tuning product selectivity, suppressing competing side reactions (especially the hydrogen evolution reaction (HER)), and enhancing CO production through the formation of *COOH intermediate [1, 2]. Tin in Cu–Sn alloys or modified surfaces tends to modulate the binding of intermediates during CO_2_RR. Specifically, low amounts of Sn (or Sn in oxidized form) can weaken the binding of *CO (the adsorbed CO intermediate), facilitating its desorption rather than further promote reduction or hydrogenation. This helps to maintain high Faradaic efficiency (FE) toward CO. On the other hand, when Sn is present in higher amounts or in certain oxidized states (SnO_*x*_), selectivity may shift toward formate (HCOO^−^) or favor HER [3]. The Cu to Sn ratio, as well as whether they form solid solutions, intermetallic compounds, or core–shell/oxide overlayers, affects both the available active sites and their binding energetics, as for example reported in Morimoto et al. [3]. At the atomic scale, several DFT studies have shown that isolated Sn motifs in Cu can tune key adsorption energetics by moderating *COOH/*CO binding (thereby promoting CO release), whereas more Sn‐rich or SnO_x_‐related environments tend to favor *OCHO stabilization, consistent with a shift toward formate [4, 5]. In addition, DFT + microkinetic studies on Cu/SnO_2−x_ interfacial models emphasize that selectivity depends sensitively on the local interfacial structure, oxidation state, and coverage, which motivates operando approaches to identify the relevant active motifs.

Despite this understanding, most insights on Cu–Sn catalyst behavior relies on ex situ characterization, assuming that the as‐prepared catalysts structure is representative of its active state. However, Cu‐based catalysts are well known to undergo structural and morphological evolution (for example by roughening, faceting) under reaction conditions [6]. Dissolution, redeposition, and in general structure and morphology changes are known to be able to fundamentally alter the nature of the active sites. Operando techniques are powerful tools, providing insights into the correlation between the catalyst structure and composition, with electrochemical activity in realistic operating device [7, 8].

Electrochemical liquid‐phase transmission electron microscopy (EC‐LPTEM) allows to visualize catalyst in real time and can reveal how materials dynamically restructure during potential or current application, thus providing insights that can guide the rational design of catalysts with improved performance [6]. Early studies showed that oxide‐derived Cu catalysts undergo rapid restructuring through dissolution and redeposition processes during CO_2_ electroreduction [9]. Buonsanti and coworkers, combining EC‐LPTEM with operando X‐ray absorption spectroscopy, demonstrated that Cu nanoparticles experience cycles of partial dissolution followed by localized redeposition, leading to dendritic growth and morphological heterogeneity that directly impact catalytic performance [9]. Tileli and colleagues, reported the nucleation of secondary Cu nanoparticles, attributed to oxidation‐induced dissolution followed by potential‐driven redeposition, thereby clarifying the degradation pathways in copper catalysts [10]. Chee et al. showed that in a conventional electrolyte for CO_2_RR (i.e., KHCO_3_), Cu_2_O nanocubes undergo extensive dissolution–redeposition, losing their cubic morphology and forming irregular nanostructures; which is recognized as evidence of copper's intrinsic instability under reaction conditions [11]. These studies highlight the ability of operando EC‐LPTEM to unravel the interplay between catalyst structure and electrolyte composition in determining stability and activity during CO_2_ reduction.

The coupling of Sn with Cu, and the formation of oxide‐derived Cu–Sn interfaces enable a synergistic interplay, making Cu–Sn oxide systems particularly attractive for studying the structure–activity relationship under operando electrochemical conditions.

In this work, we investigate the operando structural evolution of Cu_2_O–SnO_2_ (functionalized with Re) catalysts during CO_2_ electroreduction, with emphasis on the earliest reconstruction processes that determine the subsequent catalyst state. Using electrochemical liquid‐phase TEM (EC‐LPTEM) complemented by lab‐scale electrolyzer measurements, we directly visualize dissolution/reprecipitation phenomena and the stabilizing role of SnO_2_ coverage under bias. By correlating operando structural dynamics and characterization of the materials with product selectivity trends, we highlight how catalyst restructuring and interfacial evolution under realistic operation can govern performance beyond what is inferred from ex situ characterization.

## 2 Experimental Section

### 2.1 Catalyst Synthesis Procedure

The Cu_2_O–SnO_2_–Re catalyst was synthesized using an adapted procedure [12]: briefly, Cu_2_O–SnO_2_ nanocubes were obtained by wet precipitation and coordination etching. The as‐prepared nanoparticles were functionalized with vinyltriethoxysilane (VTES), which was then used to attach a rhenium (Re) molecular complex via an electropolymerization process (Cu_2_O–SnO_2_–Re), the latter acting as a cocatalyst to enhance the CO_2_ reduction to CO. In this work, the Cu_2_O–SnO_2_–Re catalyst has been characterized before, during (using an advanced operando charactrerization) and after the electrocatalytic activity in a lab‐scale electrolyzer. The choice of catalyst material, Cu_2_O–SnO_2_–Re, was strategically aligned with the initial objectives of the SunCoChem project [13], which aimed to develop a TRL5 prototype combining CO_2_ capture with photoelectrochemical conversion for local syngas production under sunlight.

### 2.2 Electrode Preparation for Lab‐Scale Electrochemical Experiments

The Cu_2_O–SnO_2_–Re gas diffusion electrode (GDE) was prepared by airbrushing a catalytic ink onto Sigracet 39BB (Fuel Cell Store) carbon paper. The ink consisted of: (i) Cu_2_O–SnO_2_–Re powder, (ii) Nafion perfluorinated resin solution (5 wt% dispersion in water and 1‐propanol, Sigma–Aldrich) as a binder and to facilitate ion transport within the electrode, and (iii) ethanol (≥99.5%, ACS reagent, Sigma–Aldrich) as the solvent and carrier for deposition. The Cu_2_O–SnO_2_–Re GDE was prepared with a geometric active area of 120 cm^2^ and a catalyst loading of ≈4 mg cm^−2^. The carbon paper sheets were placed on a heating plate to ensure complete solvent evaporation during the deposition. The electrodes were kept on the heating plate for an additional time to guarantee complete drying.

### 2.3 Electrolyzer Configuration and Operating Conditions

**FIGURE 1:**
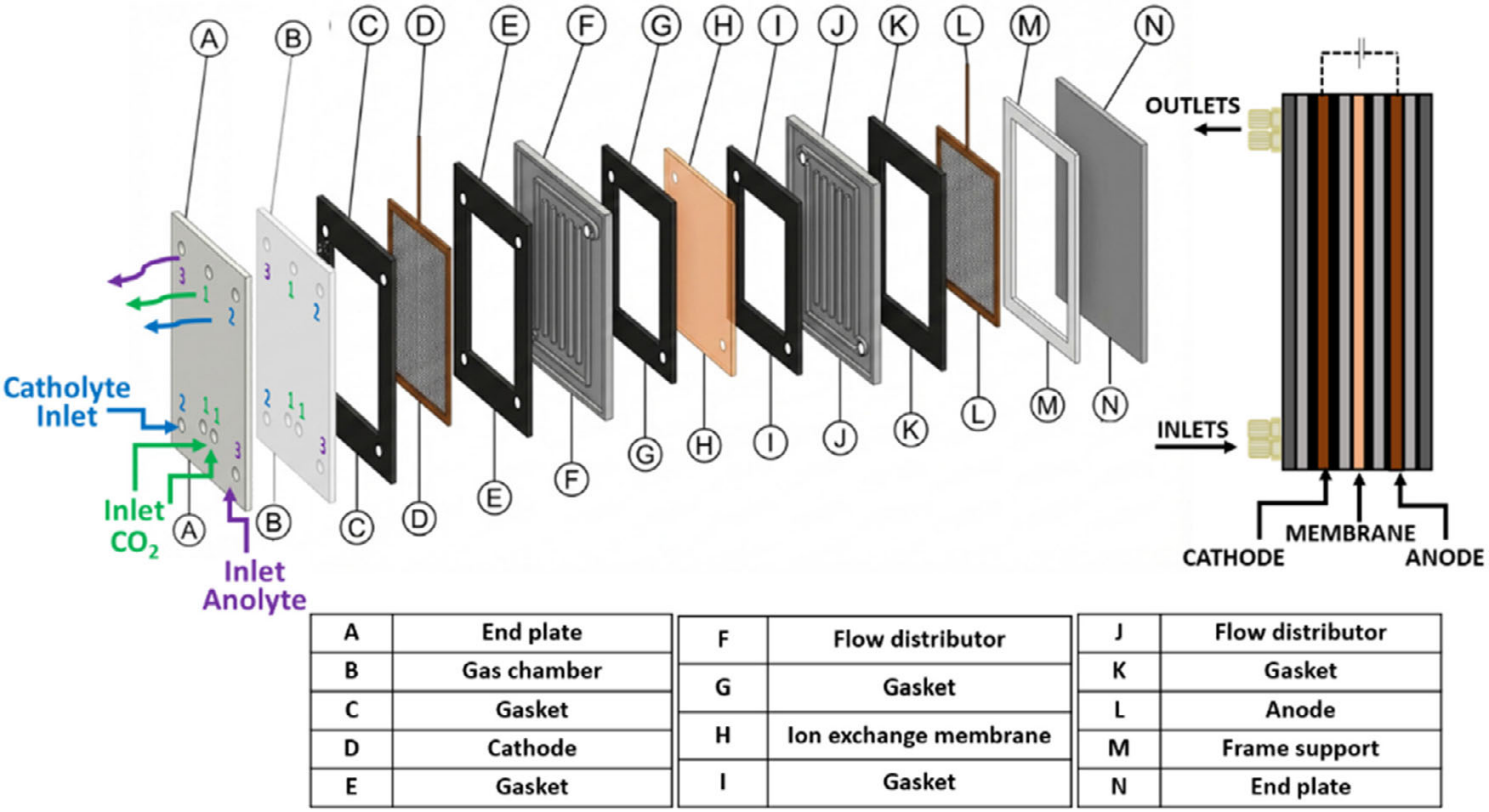
Schematic layout of the customized three‐compartment 120 cm^2^ electrolyzer.

A 120 cm^2^ electrolyzer prototype (Figure 1), manufactured by Hysytech S.r.l. with design contributions from Politecnico di Torino, was employed in the lab‐scale electrochemical investigation. The cell architecture integrates both gas and liquid manifolds within the same housing to simplify assembly and operation. As shown in the exploded view (Figure 1), the stack is mechanically compressed between two end plates (A and N). On the cathode side, a dedicated gas chamber (B) distributes the CO_2_ feed to the gas‐facing side of the cathode. Sealing and definition of the active area are ensured by a set of gaskets (C, E, G, I, and K), which also prevent cross‐leakage between the gas and liquid compartments. The working electrode (WE), a Cu_2_O–SnO_2_–Re GDE (D), is positioned between the CO_2_ gas chamber (B) and the catholyte compartment, enabling operation under gas–liquid conditions. A flow distributor (F) is placed on the catholyte side to promote uniform electrolyte distribution and stable contact with the electrode surface. In the gas chamber, a polymeric mesh is used to further improve CO_2_ flow uniformity.

An ion‐exchange bipolar membrane (H) separates the catholyte and anolyte chambers: the cation‐exchange layer faces the catholyte side, while the anion‐exchange layer faces the anolyte side. The catalytic layer of the Cu_2_O–SnO_2_–Re GDE is directly exposed to a continuous catholyte flow (0.1 M KHCO_3_). On the anode side, an additional flow distributor (J) ensures homogeneous anolyte flow (3 M KOH) and contact with the counter electrode. A Ni mesh serves as the counter electrode (L). A frame support/spacer (M) is included for alignment and mechanical stability of the assembly.

During operation, CO_2_ and the liquid electrolytes (catholyte and anolyte) are fed separately from the bottom inlets, while the corresponding outlets for gas, catholyte, and anolyte are located at the top of the cell (Figure 1). A 1 mm leak‐free Ag/AgCl (3.4 M KCl) electrode was employed as the reference electrode in the three‐electrode configuration.

The electrolytes were circulated at a rate of 60 mL/min using a peristaltic pump, with each stream connected to an external 500 mL reservoir. The CO_2_ flow rate was fixed at 90 NmL/min. The electrolyzer was run under ambient conditions, with pressure across the chambers regulated by back‐pressure controllers and monitored by pressure transducers. Inline gas product analysis was performed using a micro gas chromatograph (Agilent 990 µ GC) equipped with Molsieve 5 Å and PoraPLOT U capillary columns. Electrochemical measurements were conducted with a multichannel potentiostat (BioLogic VSP‐300) equipped with a 2 A/30 V booster. Galvanostatic measurements (chronopotentiometry, CP) were performed at current densities ranging from −6 to −20 mA cm^−2^ to evaluate the selectivity and stability of the systems.

### 2.4 Assembly of the Operando Transmission Electron Microscopy Cell

The operando experiments were performed using a MEMS‐based transmission electron microscopy (TEM) cell assembled using two commercial chips and mounted in a liquid cell holder (Poseidon Select, Protochips Inc.) (see Figure S1). The large chip features a three‐electrode setup, allowing the application of current and potential to the sample, while the small chip contains a microfluidic pattern that guides the electrolyte flow. The chips were carefully aligned to superimpose the two electron‐transparent windows (a 50 nm film of Si_3_N_4_), maximizing TEM viewing region.

The large electrochemical chip (ECT‐45CR) has a glassy carbon WE and platinum counter‐ and reference electrodes (CE, RE). The small chip (ECB‐55GF‐FM) contains a 10 µm channel patterned all around the viewing window to improve the flow of the electrolyte, as previously shown [14, 15]. The electrolyte thickness in the viewing window is determined by the spacers placed on both chips. A total thickness of nanochannel of 1 µm was selected, as it represents an optimal compromise between achieving adequate TEM resolution and accommodating the catalyst particles, with a size range of 200–500 nm, while minimizing the risk of membrane rupture or electrical short‐circuiting.

A dispersion of the Cu_2_O–SnO_2_–Re catalyst particles in ethanol was deposited onto the WE by drop‐casting using a syringe and a silicon mask to confine the material within the WE area. The assembled cell was filled with CO_2_‐saturated 0.1 M KHCO_3_ electrolyte, and the presence of liquid was verified under an optical microscope. Electrochemical measurements were conducted with a flow rate of 1200 µL/h to continuously refresh the electrolyte and remove reaction products. Prior to inserting the holder into the TEM, the absence of leakage was confirmed using a HiCUBE (Pfeiffer) pumping station. All electrochemical experiments were performed using a Gamry Reference 600+ floating potentiostat.

### 2.5 Electron Imaging

TEM imaging was performed with a Tecnai F20ST (Thermofisher, former FEI) operating at 200 kV, equipped with a Fischione high‐angle annular dark field detector and a Bruker XFlash 7 Energy Dispersive X‐ray Spectroscopy (EDS) detector. The same TEM was used to perform both operando and post mortem analysis of the sample. In addition, EDS maps were collected with a Thalos 200 (Thermofisher, former FEI) equipped with QuadX EDS.

During the operando experiments, the illumination conditions were kept fixed, particularly in scanning transmission electron microscopy (STEM) mode with a dose per frame of ≈1 e^−^ nm^−2^.

Field‐emission scanning electron microscopy (FESEM) imaging of the pre‐ and post mortem catalyst was performed using dual beam FIB‐SEM workstation (Auriga model by Zeiss), equipped with an Oxford XMAX detector for EDS. For the as‐is measurements, the Cu_2_O–SnO_2_–Re catalyst was dispersed in powder form on the carbon tape. For the post mortem analysis, the electrode after electrochemical testing was used.

Cross sections of the catalyst particles were prepared by Focused‐Ion Beam milling (FIB, ZEISS Auriga), and analyzed by FESEM. Milling was performed at 30 kV and 2 nA, then refined at 30 kV with currents of 240 and 50 pA to minimize ion induced damage, similar to procedure described previously [16]. Prior to milling, a Pt protection cap layer was deposited using an ion beam‐assisted procedure, with a Gas Injection System.

### 2.6 X‐ray photoelectron spectroscopy Characterization

X‐ray photoelectron spectroscopy (XPS) measurements were carried out using a PHI 5000 VersaProbe II spectrometer (Physical Electronics, USA) equipped with a monochromatic Al Kα X‐ray source (1486.6 eV). The X‐ray source was operated at a power of 24.78 W, and the photoelectrons were collected with a take‐off angle of 45° with respect to the sample surface. The base pressure in the analysis chamber during measurements was maintained below 10^−5^ Pa. Survey spectra were acquired in the binding energy range of 0–1200 eV using a pass energy of 187.85 eV, while high‐resolution spectra (HR) regions were recorded with a pass energy of 23.50 eV. Charge neutralization was performed using the dual‐beam charge compensation system (low‐energy electrons and Ar^+^ ions). The binding energy scale was calibrated by setting the C 1s peak of adventitious carbon to 284.8 eV. All spectra were processed and fitted using Multipak Version 9.9 software. A Shirley background was subtracted, and the core‐level spectra were fitted using mixed Gaussian–Lorentzian line shapes. Atomic concentrations were calculated from the integrated peak areas using the sensitivity factors supplied by the instrument manufacturer.

## 3 Results and Discussion

### 3.1 Characterization of As‐Prepared Cu_2_O–SnO_2_–Re Nanoparticles

**FIGURE 2:**
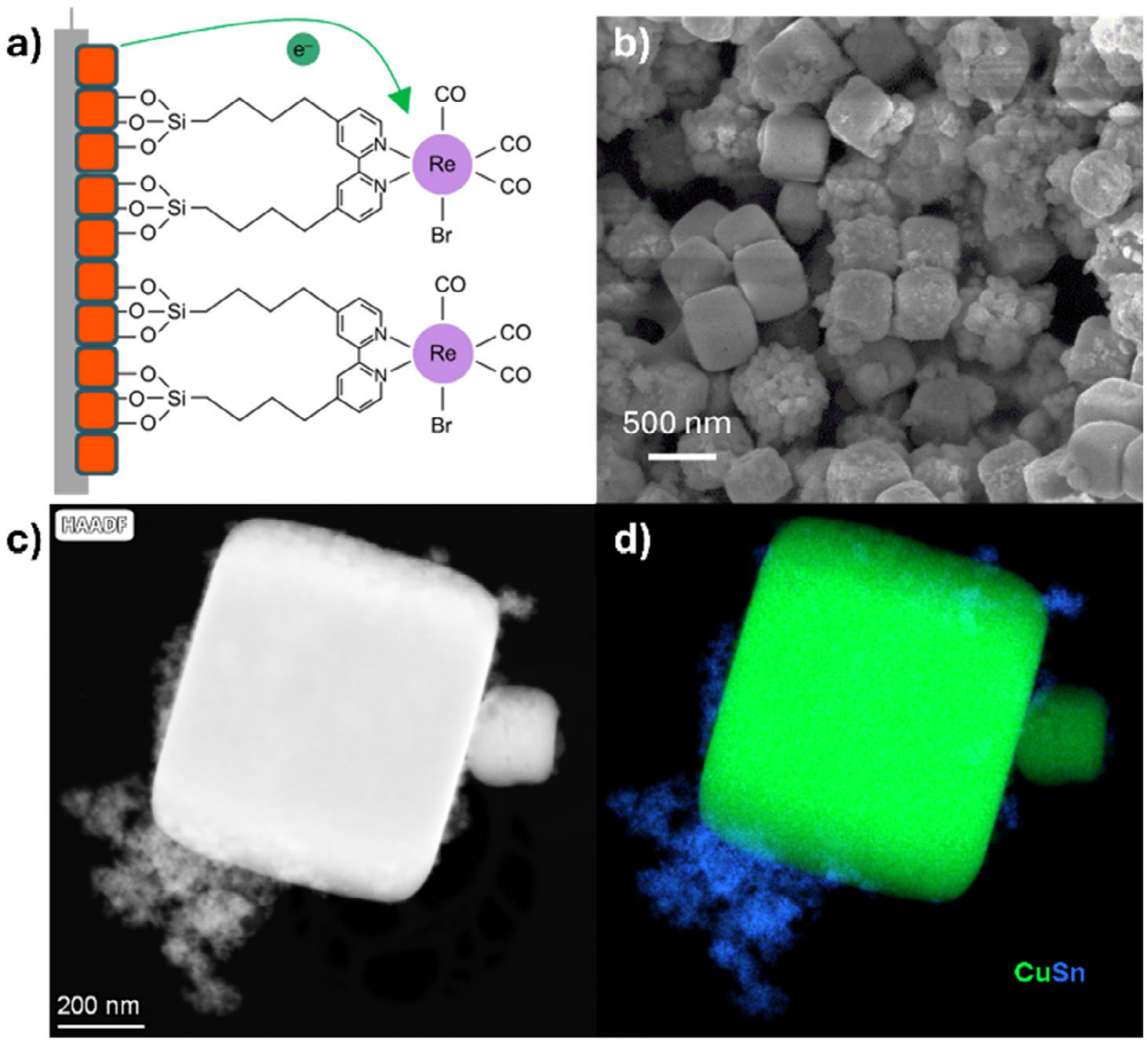
Cu_2_O–SnO_2_–Re in the as‐prepared state: (a) schematic of the chemical structure; (b) FESEM image; (c) HAADF STEM image, and (d) EDS mapping showing the distribution of Sn (blue) and Cu (green) in the particles.

In Figure 2a, the schematic of the Cu_2_O–SnO_2_–Re catalyst structure, as described in the experimental section, is shown. It consists of a core Cu_2_O cubic crystal (orange color) covered by a thin SnO_2_ shell (blue surrounding line), onto which vinyltriethoxysilane and the rhenium molecular complex are anchored.

Owing to this core–shell like design, the catalyst predominantly exhibits a cubic morphology, with cube sizes ranging from ≈200 nm to 700 nm. In addition to these well‐defined cubes, agglomerates of smaller nanoparticles are also experimentally observed, as evidenced by the FESEM and STEM images shown in Figure 2b,c. Preliminary EDS investigations indicate that the cubes are mainly composed of Cu, while the nanoparticles contain Cu, Sn, and O. For clarity, Figure 2d reports only the Cu and Sn distributions, as oxygen is detected throughout the sample [12]. The catalyst consists of Cu(I) oxide and Sn (IV) oxide, as reported in previous work [12].

**FIGURE 3:**
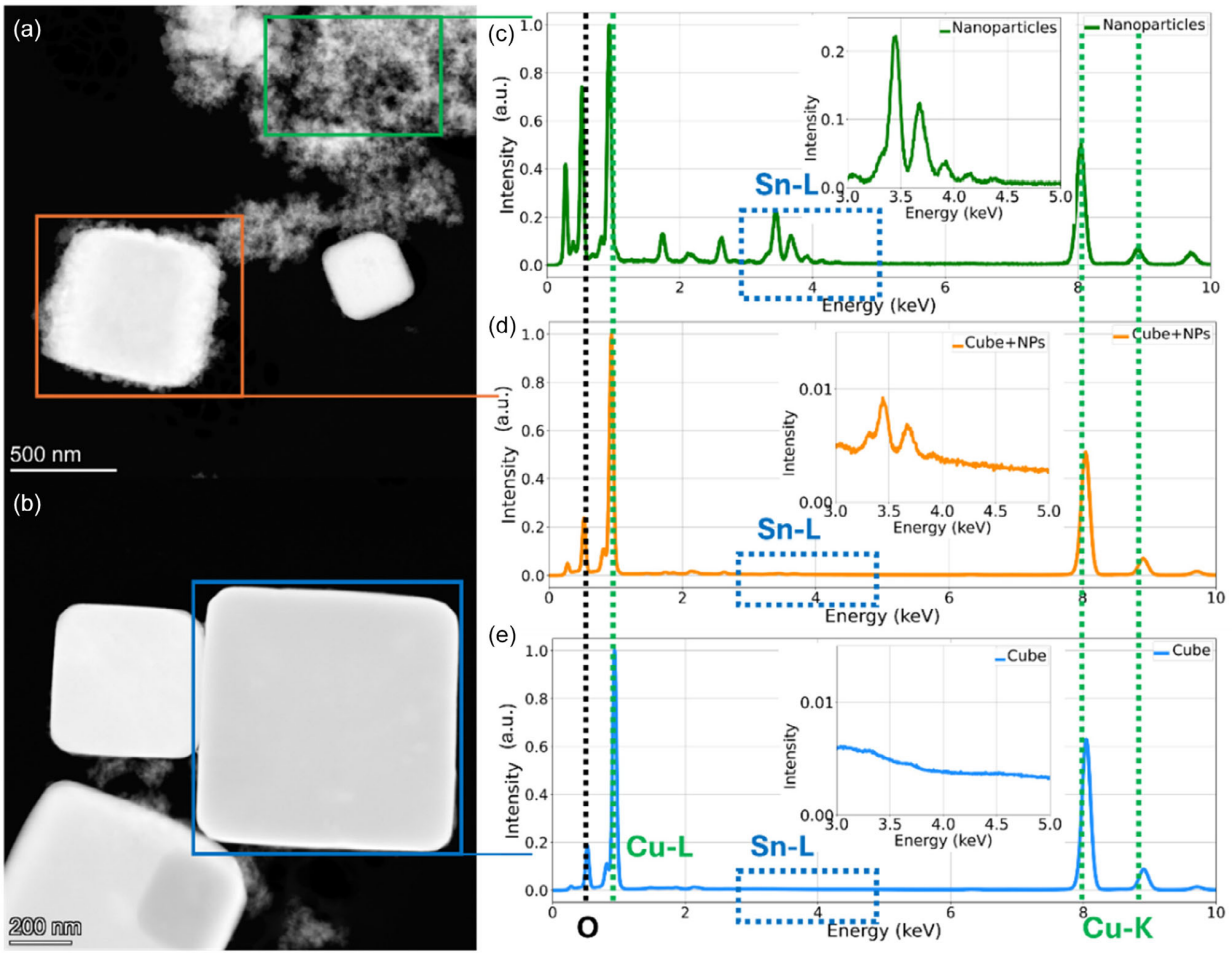
STEM HAADF images (a,b) with corresponding EDS spectra (c–e), showing the chemical composition of the different types of particles present in the catalyst. Insets in EDS spectra (c–e) show a zoom on the region of the L spectral line of Sn, between 3 keV and 5 keV. All spectra have been normalized to the the Cu‐L line intensity.

To further investigate the nanostructure composition, EDS has been collected on different types of particles. In Figure 3a, a cube displaying nanoparticle aggregates adhering to its surface is highlighted, whereas in Figure 3b, a cube with a comparatively smoother surface is shown. Aggregates of nanoparticles can be identified both on the surface of the cubes and in their vicinity (Figure 3a). The EDS spectrum of the nanoparticle aggregates (Figure 3c) displays pronounced and sharp peaks corresponding to the L and K lines of Cu and the L lines of Sn (between 3 and 4.5 keV, as shown in the inset of Figure 3c), evidencing that these nanoparticles contain both Cu and Sn (in atomic concentration of 34.1% and 9.6%, respectively). In contrast, the rough Cu_2_O cubes located near the nanoparticle aggregates exhibit strong Cu peaks, while the peaks associated with Sn appear with markedly lower intensity (inset of Figure 3d). The EDS semiquantitative analysis suggests a high concentration of Cu (66%), while Sn is present only in traces (0.4%), indicating that Sn is probably confined to the thin surface‐associated coating the cubes, which explains its lower relative abundance compared to Cu. To verify this hypothesis, smoother cubes not covered by nanoparticles were also analyzed. The EDS spectrum shown in Figure 3e reveals the absence of the Sn L‐line peaks (between 3 keV and 4 keV), confirming that these cubes do not contain detectable amount of Sn. Rhenium is present only in trace quantities, as previously reported in [12], below the detection limit of EDS analysis.

In order to determine whether the uncovered cubes completely lack the Sn shell, or whether the shell is sufficiently thin to remain undetected by EDS analysis, the catalyst was examined in cross‐section. Cross‐sections of the as‐prepared electrode were prepared by FIB and analyzed by FESEM. The cross‐sectional image shown in Figure S2 reveals that a large fraction of the cubes is covered by a granular SnO_2_ shell. The shell thickness varies among different cubes and locally within the single cubes, and a continuous coverage is not always observed. In addition, as mentioned previously, some Cu_2_O cubes are completely missing the shell.

To gain insight into the surface composition of the catalyst and the oxidation states of the constituent elements, XPS measurements were performed on the as‐prepared electrode. The results indicate that the catalyst contains predominantly Cu(I), with a minor contribution from Cu(II), as evidenced by the high‐resolution spectra of the Cu 2p region and the Cu Auger region (see Figures S3 and S4, details on XPS analysis in Supporting Note 1). XPS analysis also confirms the presence of SnO_2_, with its characteristic Sn 3d_5/2_ peaks shifted toward lower binding energy, which may indicate strong interfacial interactions within the Cu–O–Sn system (see Figure S5). Given the high surface sensitivity of XPS, this technique was used to estimate the surface Cu/Sn ratio, which is expected to be higher than the bulk ratio, as Sn forms the shell of the particles and is therefore mainly located at the surface. Indeed, the Cu:Sn ratio calculated from the high‐resolution XPS spectra is 2.4:1, which is significantly lower than the average Cu:Sn ratio of 22:1 obtained from EDX, reflecting the surface‐enriched nature of Sn. XPS measurements also reveal the presence of a small amount of rhenium in the sample, as shown in Figure S6, thus confirming the expected structure of the catalyst.

### 3.2 Operando TEM to Monitor the Behavior of the Catalyst Under CO_2_RR Conditions

Operando EC‐LPTEM experiments were performed to investigate the catalytic behavior and stability of the Cu_2_O–SnO_2_–Re system under reaction conditions. STEM imaging was employed to monitor the catalyst during chronopotentiometric operation (i.e., at a fixed applied current density). The application of a cathodic current induces gas bubble formation, due to the coalescence of gaseous products (mainly due to CO_2_RR and water splitting reactions) formed at both the WE and the counter electrode (CE) [14, 17]. Gas bubble formation can cause disruption during electrochemical characterization, including electrode dewetting with consequent blockage of electrochemical measurements, ultimately leading to the failure of the experiment. To minimize this effect, some precautions have been taken, including a catalytically inert current collector (glassy carbon), a flow‐optimized cell, and an increased flow rate to expedite the removal of unwanted products [14]. For the Cu_2_O–SnO_2_–Re catalyst, the electrochemical reaction expected to occur at the WE (CO_2_RR) produces gaseous products (CO, H_2_ [12]), and consequently, gas bubble formation cannot be entirely avoided in these experiments.

In the first operando experiment, conditions were chosen to maintain the cell fully filled with electrolyte throughout the experiment, avoiding interference from gas bubbles. For this reason, a slightly negative current density of −2 mA cm^−2^ (corresponding to −50 nA in the EC‐LPTEM cell; WE area: 2500 µm^2^) was applied continuously for 50 min. Under these conditions, the CO_2_RR proceeds at a sufficiently slow rate to permit the continuous removal of gas bubbles by electrolyte flow. Chronopotentiometry was preferred over chronoamperometry to better match the experimental conditions of lab‐scale testing. This current density is the first point of a series of increasingly higher electrochemical stimulations, and it was chosen to assess that the experimental setup is able to sustain such stimulation.

**FIGURE 4:**
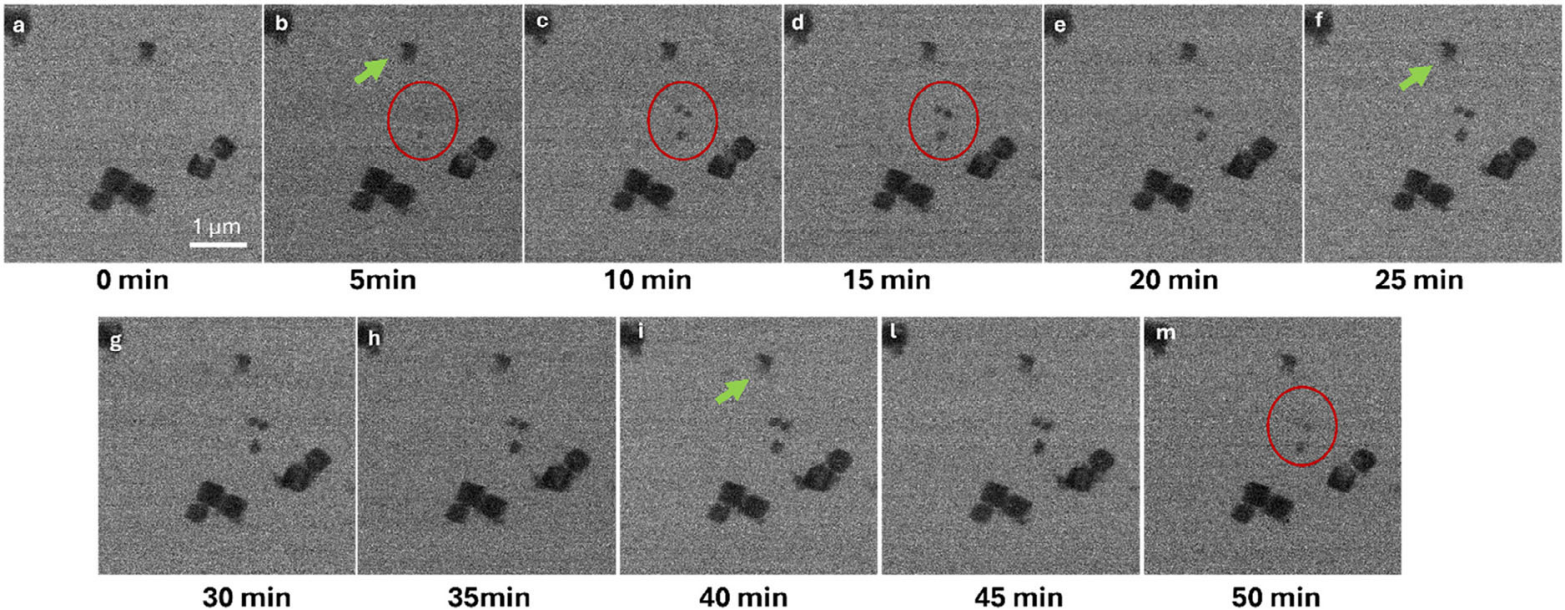
Cu_2_O–SnO_2_–Re catalyst under working conditions. Operando STEM images of the catalyst during a CP of 50 min, applying −2 mA cm^−2^ current density, showing dissolution of as‐prepared particles and reprecipitation of new ones.

Figure 4 presents the temporal morphological evolution of the catalyst under chronopotentiometric conditions. Within the first 15 min of operation, the formation of three small nanoparticles was observed (Figure 4b–d,m, red circles). These particles rapidly reached a steady morphology and remained stable throughout the remaining 35 min of the experiment. These nanoparticles likely originated from partial dissolution and subsequent redeposition of pre‐existing material under electrochemical stimulus (Figure 4, green arrow), consistent with dissolution–redeposition phenomena previously reported for Cu‐based oxide catalysts [9, 11].

The original design of the Cu_2_O–SnO_2_–Re catalyst aimed to prevent degradation phenomena during current application. Although the Cu_2_O core is known to be susceptible to electrochemical dissolution, the surrounding SnO_2_ shell, which is known to be more oxidation‐resistant, was expected to provide protection. However, the EC‐LPTEM observations revealed that some particles underwent dissolution under electrochemical bias, whereas others remained morphologically stable over time. This behavior is consistent with the presence of Cu_2_O cubes with an incomplete or missing shell of SnO_2_, as observed in the catalyst cross section (see Figure S2). These particles are more prone to dissolution, while the particles with the tin oxide shell and/or those covered with Sn nanoparticles preserve their morphology during the electrochemical stimulation.

The operando characterization thus indicates that cubes with complete SnO_2_ shell or coverage with Sn‐containing nanoparticles maintain their well‐defined cubic morphology, while those exhibiting incomplete or missing SnO_2_ coverage tend to dissolve and subsequently recrystallize. These findings confirm the protective role of tin oxide, which enhances the structural stability of the underlying copper phase during CO_2_ electroreduction.

Furthermore, operando EC‐LPTEM analysis revealed that particles undergoing dissolution tend to reprecipitate, forming smaller nanoparticles nearby. To validate this behavior and assess its dependence on electrochemical parameters, systematic measurements were performed at progressively higher current densities. These experiments enabled clearer observation of dissolution–redeposition phenomena, which become more pronounced at higher current densities.

**FIGURE 5:**
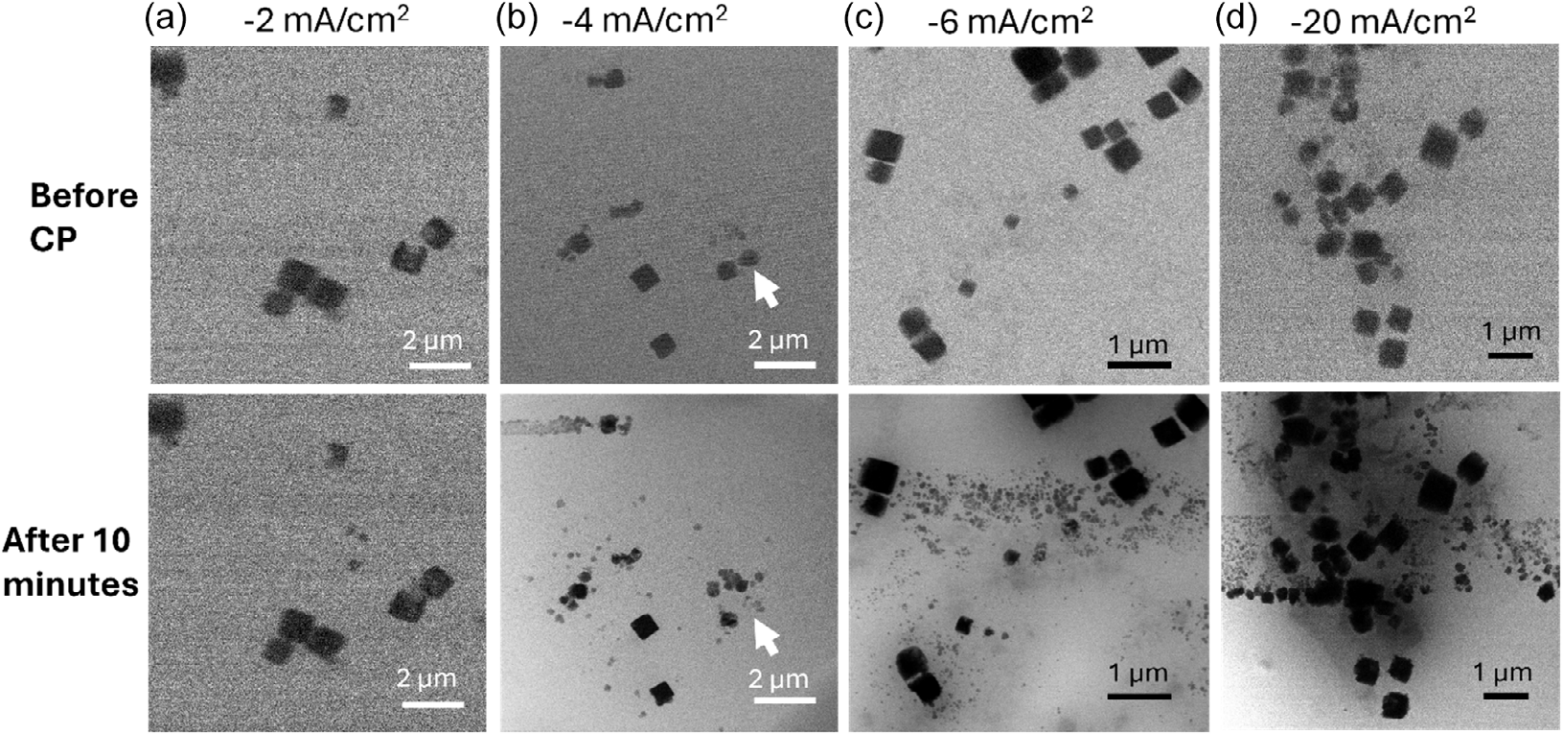
STEM images of the Cu_2_O–SnO_2_–Re catalyst under different current densities; upper row: before chronopotentiometry (CP); lower row: after 10 min of current application.

The stability of the Cu_2_O–SnO_2_–Re catalyst was therefore assessed at current densities of −4, −6, and −20 mA cm^−2^, in addition to −2 mA cm^−2^, to match conditions used in laboratory‐scale testing. Figure 5 reports TEM images of the catalyst before current application (upper row) and after 10 min of chronopotentiometry (lower row). At −2 mA cm^−2^, as discussed earlier, only a few nanoparticles nucleated, the cell remaining fully filled with electrolyte, consistent with previous observations.

When higher current densities were applied, the potential shifted toward more negative values, accelerating electrode kinetics and gas evolution. Owing to the limited electrolyte volume in the EC‐LPTEM cell, the generated gas rapidly coalesced into bubbles. Nevertheless, enhanced electrolyte flow, provided by flow management small chip, enabled the experiment to continue under electrochemical bias without dewetting of the WE, thus guaranteeing ionic contact. Under these conditions, the system operated in the so‐called *thin‐film electrolyte* condition [18]. In this regime, formed gas bubbles fill most of the cell volume, while leaving a much thinner liquid layer, which is estimated to be in order of 100 times thinner, on the catalyst surface [18]. Although under these conditions the mass transport properties are altered, with possible local pH changes, ionic contact between the electrodes is preserved. Indeed, the high electrolyte flow rate employed, enabled by the optimized cell geometry, ensures the continuous supply of fresh electrolyte, thereby maintaining ionic contact between the electrodes and the availability of dissolved CO_2_. As a positive outcome of this condition, the *thin‐film electrolyte* allows sharper TEM contrast and the appearance of localized dark regions associated with electrowetting near conductive areas (Figure 5, lower row) [19].

The morphological evolution of the Cu_2_O–SnO_2_–Re catalyst at increasing current densities follows a consistent trend. Some cubic particles dissolve (see Figure S7), while new clusters of nanoparticles nucleate in their proximity. The persistence of undamaged cubes supports the hypothesis that a complete SnO_2_ coverage effectively protects the Cu_2_O core from electrochemical dissolution. A clear correlation with current density emerges: higher applied current densities result in a larger number of newly formed smaller nanoparticles (Figure 5a–d).

At −2 mA cm^−2^, only three large nanoparticles appeared, slowly forming over 15 min, suggesting that mild conditions favor particle growth over nucleation [20]. At −4 mA cm^−2^, nucleation became more frequent while individual growth remained limited, suggesting accelerated dissolution–redeposition dynamics. A representative case is highlighted in Figure 5b (white arrow), where the complete dissolution of a Cu_2_O–SnO_2_–Re crystal coincides with the growth of a nearby cluster, implying local catalytic material redistribution. This process is intensified at −6 mA cm^−2^ (Figure 5c), where numerous smaller nanoparticles clustered around pre‐existing cubes, while at −20 mA cm^−2^ (Figure 5d) nucleation became extremely abundant, with newly formed nanoparticles aligning in the near proximity to the glassy carbon WE edge (which does not show contrast in respect to the background due to its low atomic number), an area of enhanced electric field typical of microelectrode systems [21, 22].

These findings demonstrate that the Cu_2_O–SnO_2_–Re catalyst undergoes concurrent dissolution and renucleation under electrochemical bias, with the extent of transformation scaling with current density. Results support the protective role of SnO_2_, as particles with apparent complete shell or nanoparticles coverage remain structurally stable, whereas partially uncovered cubes dissolve and redeposit material nearby. This behavior highlights the dynamic nature of Cu‐based catalysts under CO_2_RR conditions and underscores the importance of stabilizing the active phase during operation.

**FIGURE 6:**
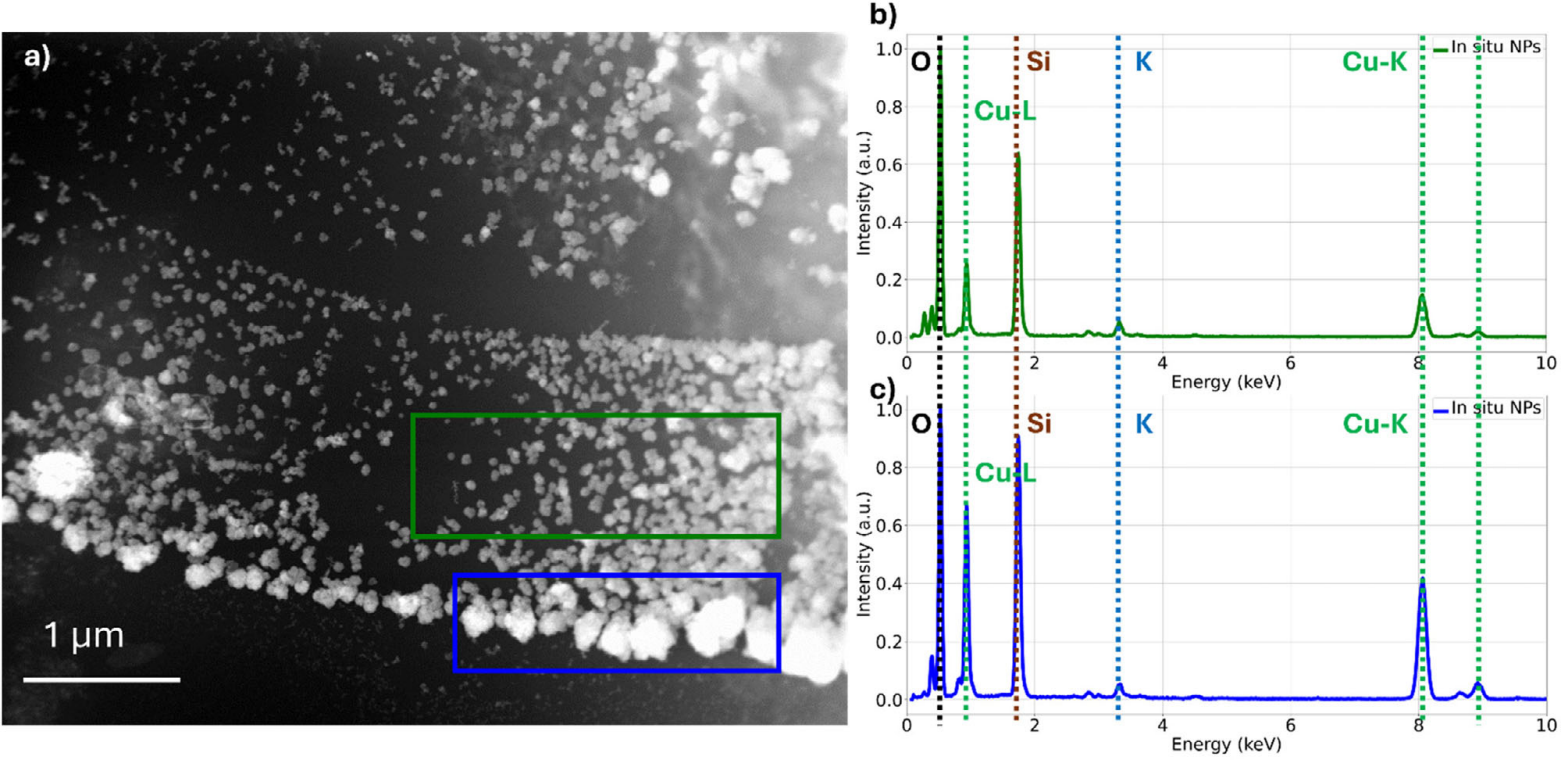
In situ STEM image (a) of newly deposited nanoparticles in the EC‐LPTEM cell, in the electrolyte thin film condition, with their respective in situ EDS (b,c).

To assess whether the redeposited particles retained the same composition as the original catalyst structures, in situ EDS analysis was conducted under *thin‐film electrolyte* condition. This configuration was selected to enhance the signal from the catalyst while minimizing interference from the liquid phase. Measurements were performed after chronopotentiometry at −20 mA cm^−2^, focusing on the nanoparticles formed during electrochemical operation to elucidate their chemical nature. Spectra were collected from both small and large recrystallized particles (Figure 6), and in all cases a consistent chemical composition was observed. This finding indicates that the nanoparticles, regardless of the size, originated from the same source and likely nucleated concurrently.

The EDS spectra indicate that copper is the only element specifically associated with the redeposited nanoparticles. Pronounced contributions from oxygen, silicon, nitrogen, and potassium are also observed, however these arise from experimental setup and surrounding environment. Oxygen from the electrolyte and oxide species, silicon and nitrogen from the Si_3_N_4_ window, and potassium from the electrolyte, are therefore all expected under these conditions. This indicates that the nanoparticles derive from dissolution of the Cu_2_O cubes into the electrolyte *via* formation of transient solution‐phase Cu(I) species (e.g., Cu(I)–OH and/or other Cu(I) complexes depending on local electrolyte composition and potential), which are subsequently reduced and redeposited under cathodic bias as Cu^0^, yielding the observed nanoparticles. Such a dissolution–redeposition reconstruction pathway is now well documented for Cu and oxide‐derived Cu catalysts during the initial stages of CO_2_RR and during Cu‐oxide reduction, including direct operando/in situ microscopy evidence of partial dissolution followed by localized redeposition, and complementary operando analytics identifying the dissolved transient intermediates as Cu(I) [23]. Because our present measurements do not directly resolve aqueous Cu speciation, we avoid assigning a unique intermediate and instead refer to a Cu(I) transient pool, consistent with recent analyses emphasizing the broad relevance and early onset of dissolution–redeposition across Cu morphologies [24, 25].

Although the presence of oxygen in the surrounding medium prevents a definitive distinction between metallic copper and copper oxides in the EDS spectra (Figure 6b,c), the application of a reducing current strongly suggests that the initially present Cu(I) species are reduced to metallic Cu. This interpretation is consistent with previous literature reporting the dissolution and redeposition of copper oxides as metallic Cu during electrochemical reduction [11].

Importantly, no Sn signal (expected between 3 keV and 4 keV) was detected within the redeposited particles, demonstrating that Sn remains stable under the applied electrochemical conditions and does not take part in the dissolution–reprecipitation process. This observation reinforces the proposed protective role of the SnO_2_ shell, which effectively preserves the structural integrity of the underlying Cu_2_O core and validates the concept behind the catalyst design.

To assess the intrinsic stability of the Cu_2_O–SnO_2_–Re catalyst in the electrolyte and under electron beam exposure, some particles were intentionally deposited on the electron‐transparent window adjacent to the WE (see Figure S8). Verifying their stability is essential to exclude the possibility that the structural transformations observed during operando measurements arise from other types of interactions, such as electrolyte–catalyst interaction or radiolysis.

Because the electron beam can significantly influence material transformations during operando TEM experiments [26, 27], special care was taken to minimize irradiation effects. STEM images were therefore acquired at regular intervals rather than continuously, while the rest of the time the electron beam was blanked. This approach minimized the total electron dose per frame (~1 e^−^ nm^−2^) and was systematically applied throughout all experiments. To validate this strategy to mitigate radiolysis effect, these control particles were exposed to both the electrolyte and the electron beam under identical environmental conditions as those of the active catalyst, except that they were not subjected to electrochemical biasing, being located outside the WE area. These results confirm that the structural modifications previously observed in the catalyst originate from electrochemical stimulation rather than from the liquid environment or beam‐induced effects. Furthermore, the consistent correlation between the extent of catalyst transformation and the applied current density reinforces the conclusion that, under the present experimental conditions, the influence of the electron beam is negligible compared to that of the electrochemical driving force.

### 3.3 Electrochemical Performance at Lab Scale

Electrochemical performance tests of the Cu_2_O/SnO_2_–Re catalyst were first performed in a GDE setup, using a 10 cm^2^ electrode, allowing the reference electrode to be placed close to the cathode (additional details on the 10 cm^2^ setup are provided in the Supporting Note 2). In such a setup, the influence of the Re complex on catalytic behavior can be clearly observed, allowing its contribution to be distinguished from mass‐transport and fluid‐dynamic limitations, which tend to become more prominent as the scale of the electrochemical cell increases. As shown in Figure S9 , the presence of Re complex has minor impact on the CO_2_ reduction selectivity compared to the bare Cu_2_O/SnO_2_ catalyst. Within a current density range from −5 to −20 mA cm^−2^, the catalyst achieved a CO/H_2_ ratio of 2–6, with a CO FE of up to 60% and an average formate FE of 20%. Under these conditions, the bare Cu_2_O/SnO_2_ catalyst follows a trend consistent with that including Re complex, indicating a comparable electrocatalytic response. However, the benefit of Re complex as a cocatalyst was apparent from the lower potential observed across test sets. Figure S10 in the Supporting Information clearly shows that, in the presence of the Re complex, the working potential shifts to lower values at all current densities (see Table S1 for additional details).

**FIGURE 7:**
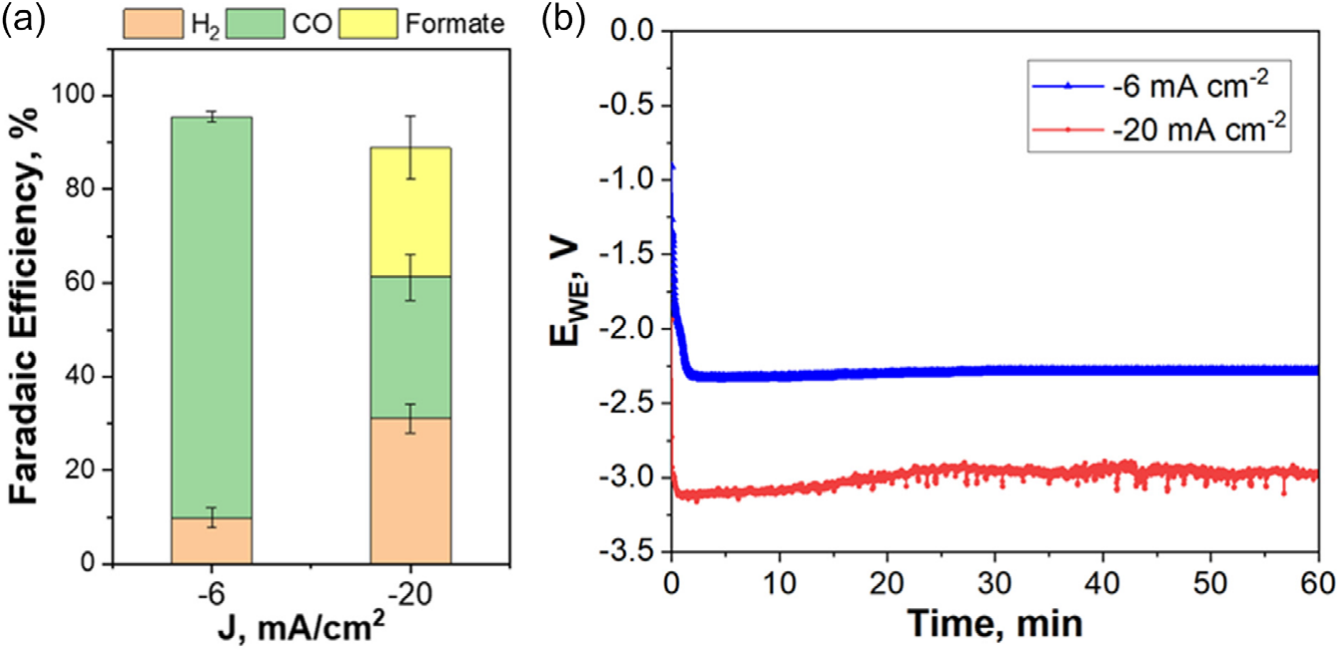
(a) Faradaic efficiencies for CO, H_2_, and formate measured on a 120 cm^2^ Cu_2_O–SnO_2_–Re GDE in 0.1 M KHCO_3_ at constant current densities of −6 and −20 mA cm^−2^. Error bars represent the standard deviation of the Faradaic efficiencies (from *n* = 3 replicate runs). (b) Cathode potential (E_WE_) recorded as a function of time during chronopotentiometry at −6 mA cm^−2^ (blue) and −20 mA cm^−2^ (red).

Based on these results, the effect of current density on the catalytic performance of the Cu_2_O–SnO_2_–Re catalyst was subsequently evaluated on electrode area of 120 cm^2^. Galvanostatic measurements of ≈1 h were carried out in a range from −6 mA cm^−2^ to −20 mA cm^−2^, as shown in Figure 7 (see Table S2 for additional details). This range was chosen in line with the objectives of the SunCoChem EU Project, which aims to deliver a solar‐powered photoelectrochemical device to the European Chemical Industry for the sustainable production of oxo‐products by coupling it with a hydroformylation or carbonylation process [13]. Figure 7a illustrates the effect of current density on product selectivity. At lower current density (−6 mA cm^−2^), the catalyst shows a high Faradaic efficiency (~90%) toward CO, which can be associated with the presence of exposed Cu_2_O and/or subsurface oxygen on the catalyst surface [28]. Such Cu^+^‐rich sites are reported to stabilize the *COOH intermediate, thereby favoring CO formation while suppressing H_2_ evolution [29].

Nonetheless, as the current density increases (−20 mA cm^−2^), hydrogen evolution becomes more prominent. At the same time, the CO Faradaic efficiency decreases, enabling a CO/H_2_ ratio of 1.2, which was a target condition in the project for the subsequent hydroformylation of Limonene to Limoxal. This selectivity change could be due to reduced CO_2_ transport to the catalyst surface, resulting in lower CO_2_ availability under these current density conditions [30]. Indeed, both CO production and the associated requirement for counter‐diffusion are expected to rise with current density, ultimately leading to a decline in CO_2_ mass transport at the electrode–electrolyte interface. In parallel, operando TEM analysis suggests that Cu_2_O domains may undergo dissolution and recrystallization into smaller metallic Cu nanoparticles. These newly formed Cu^0^ particles are possibly defect‐rich and under‐coordinated, favoring proton reduction and contributing to the increase in H_2_ production. This observation is consistent with the literature on oxide‐derived Cu, where the loss of Cu^+^ species and restructuring into metallic Cu domains results in a drop in CO selectivity [31].

Of particular interest, however, is the marked shift in selectivity between CO and formate: CO decreases from ~90% FE at −6 mA cm^−2^ to ~40% FE at −20 mA cm^−2^, while formate emerges as a significant product. This behavior suggests that multiple catalytically relevant motifs coexist and respond differently to current density. At low current densities, where operando TEM suggests limited restructuring, the higher availability of CO_2_ likely enables its conversion to CO on Cu/Cu^+^‐rich regions (and/or oxygen‐containing motifs), which have been widely discussed in the oxide‐derived Cu literature as influencing the binding of early intermediates and suppressing competing H_2_ formation [32]. As the current density increases, extensive restructuring occurs, as demonstrated by operando EC‐LPTEM, and may lead to a pronounced separation between Cu/Cu–O and Sn/Sn–O domains, together with the formation of new Cu–Sn interfacial regions. Literature DFT and DFT–microkinetic studies on Cu–Sn systems (including Cu/SnO_2−x_ interfacial models) show that changes in interfacial composition/oxidation state and coverage can significantly modulate the relative stabilization of carboxyl (*COOH/*CO) versus formate‐associated (*OCHO) intermediates, thereby shifting selectivity between CO and formate [32, 33]. In addition, operando */*in situ studies emphasize that oxide‐derived catalysts can undergo substantial transformations under testing conditions, highlighting that the catalytically active interface is dynamic rather than static. Consistent with these reports, in our system, the combined effects of (i) reduced CO_2_ mass transport (Supporting Note 3, Figure S11), (ii) loss of Cu^+^‐rich domains through dissolution–recrystallization into smaller Cu‐rich particles, and (iii) increased contribution of SnO_2_‐related sites and Cu–Sn interfacial motifs under high‐current operation, provide a coherent explanation for the observed current‐density‐dependent product distribution [30, 34]. Furthermore, the higher local pH expected at elevated current densities is less favorable for CO production via *COOH but can be more compatible with formate formation via *OCHO‐associated pathways [35].

Atomistic modeling (e.g., DFT adsorption energies/electronic structure) would provide a valuable tool for quantifying the *COOH versus *OCHO competition on our catalyst. In the present work, however, operando TEM evidence indicates that the oxide‐derived Cu–Sn interface dynamically evolves with current density and time (reconstruction, changing local coordination, and changing relative exposure of Cu‐rich vs. SnO_*x*_‐rich motifs). Within this dynamic framework, our study provides a strong experimental basis for a literature‐grounded mechanistic interpretation. Building on these insights, operando ‐informed DFT and microkinetic modeling emerge as a particularly promising avenue for future work to capture the full complexity of the active interface.

In situ EC‐LPTEM insights show that major morphological changes happen within the first few minutes of operation (Figures 4 and 5). Therefore, the 1‐h constant‐current test is designed to capture representative early‐stage reconstruction behavior central to this study. While EC‐LPTEM is inherently not suited for hundreds‐thousands of hours durability testing, we provide additional durability context at a practical scale: stable operation is demonstrated over >5h at −6 mA cm^−2^ on a 120 cm^2^ Cu_2_O/SnO_2_–Re GDE in 0.1 M KHCO_3_ (see Figure S12). Extended durability tests under relevant conditions (with changes in operating parameters) have also been performed and will be reported in a dedicated follow‐up publication.

### 3.4 Comparison Between Lab Scale and EC‐LPTEM Cell

**FIGURE 8:**
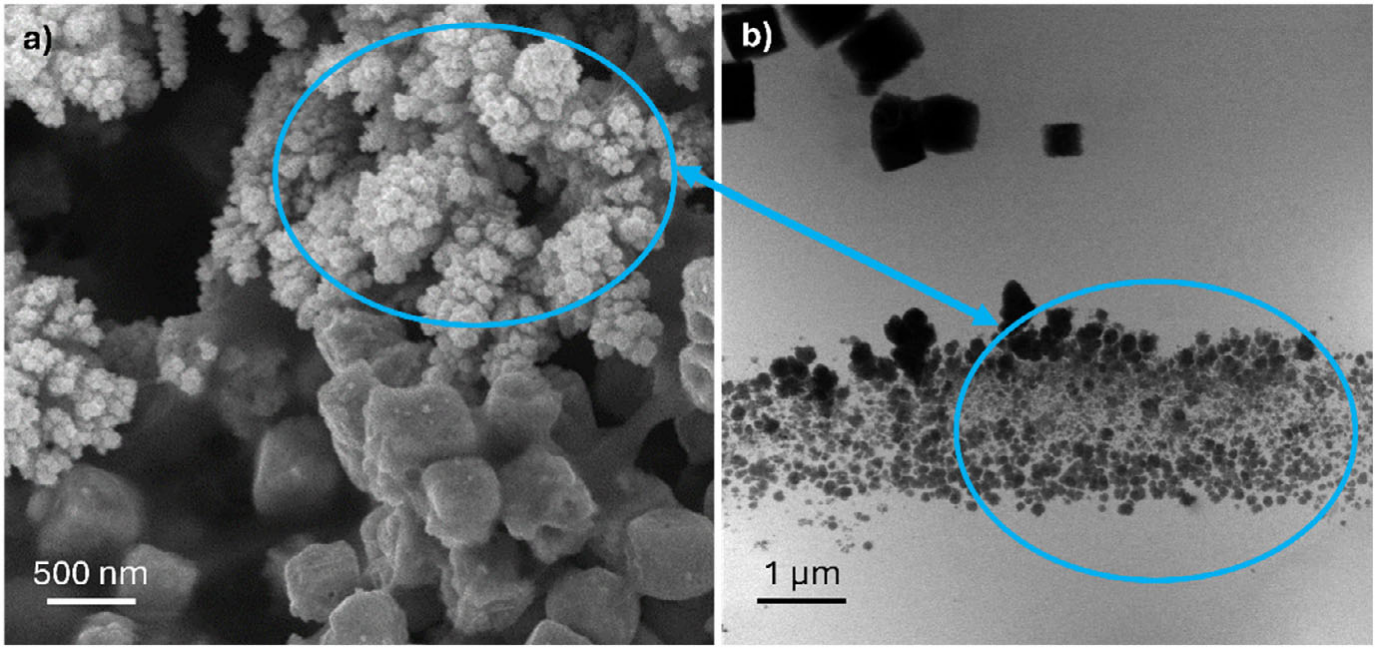
(a) FESEM of the tested lab‐scale electrode and (b) operando STEM (EC‐LPTEM cell WE) comparison of Cu_2_O–SnO_2_–Re, showing cubes and nanoparticle aggregates.

To directly correlate the observed morphology and structural changes observed during operando EC‐LPTEM with catalyst modification during conventional electrochemical testing, post mortem FESEM characterization of the Cu_2_O–SnO_2_–Re catalyst (Figure 8a) was compared with operando STEM acquired after electrochemical stimulation (Figure 8b).

The FESEM image of the tested lab‐scale electrode reveals the coexistence of well‐defined cubic particles and clusters of smaller nanoparticles. The morphology of nanoparticle clusters highlighted by the blue circle in Figure 8a closely resembles the large aggregates observed under the highest current density condition during the EC‐LPTEM experiments (Figure 8b). The aggregates observed by FESEM reveal a hierarchical architecture composed of densely packed particles. An analogous structure is observed during operando TEM, with redeposited nanoparticles forming extended accumulations and chains that align along the electrode surface in particular close to its edge, in the highest current density regions.

Complementary post mortem EDS analyses were performed to further investigate the oxidation state of copper after CO_2_RR. Top‐view measurements show a decrease in relative oxygen content, with the O/Cu atomic ratio decreasing from 0.7 in the as‐prepared electrode to 0.3 after electrochemical testing. The cross section images of pristine and tested electrode (Figure S13) show the morphological changes related to reconstruction during CO_2_RR, including the formation of nanoparticles with a different morphology, present between and around the original cubes. Cross‐sectional EDS analyses (shown in Figure S14) indicate that the redeposited nanoparticles exhibit a lower oxygen content (O/Cu = 0.16) than the Cu_2_O cubes (which also underwent the CO_2_RR) (O/Cu = 0.32), consistent with the formation of more reduced copper species under operating conditions. While the as‐prepared electrode shows an average Cu:Sn atomic ratio of ~22:1, post mortem EDS analysis of the tested electrode was performed both on entire electrode and localized on newly formed particles, and shows a change in composition, as summarized in Table S3 in Supporting Information. The average Cu:Sn ratio increases to ~58:1 due to the preferential dissolution and reprecipitation of copper.

The presence of metallic Cu in the tested electrode was confirmed by XPS measurements. In particular, the Cu Auger region exhibits two shoulders around the main peak, which are characteristic of metallic Cu, as shown in Figure S4 (see also Supporting Note 1). In contrast, Sn does not undergo a change in oxidation state after electrochemical testing and remains in the form of SnO_2_ (see Figure S5). High‐resolution XPS spectra of Cu and Sn also show an increase in the Cu:Sn ratio, from 2.4:1 in the as‐prepared electrode to 6:1 in the tested electrode. This trend is in accordance with the EDS analysis and supports the hypothesis of preferential Cu reprecipitation. At the same time, it suggests that the remaining SnO_2_ is still preferentially located at the surface, indicating that the SnO_2_ shell remains stable under electrochemical conditions. Post mortem analysis of the tested electrode also reveals the presence of rhenium, which appears to remain stable after electrochemical testing (see Figure S6).

This morphological correspondence demonstrates a strong consistency between the results obtained from lab‐scale electrochemical cells and those recorded within the microelectrode environment of the EC‐LPTEM setup. Despite inherent differences in geometry, scale, and electrolyte confinement, the operando TEM experiments successfully reproduced the structural and compositional evolution observed under conventional testing. These findings confirm that EC‐LPTEM provides a reliable and representative means of probing the morphological dynamics of the Cu_2_O–SnO_2_–Re catalyst during electrochemical operation.

## 4 Conclusion

In this work, the Cu_2_O–SnO_2_–Re catalyst for CO_2_RR was investigated by means of operando EC‐LPTEM. The catalyst was examined under cathodic current to evaluate its structural and morphological evolution under realistic operating conditions. Its rational design is based on a Cu_2_O core coated with a protective SnO_2_ shell, intended to enhance stability by mitigating degradation induced by the electrolyte and applied potential.

Under electrochemical stimulation, the observed dynamic behavior is clearly correlated with the extent of Sn coverage. Catalyst particles lacking complete SnO_2_ coverage experienced dissolution and subsequent recrystallization, whereas cubes with SnO_2_ shell and/or Sn‐containing nanoparticles coverage remained stable and retained their original morphology. A series of experiments performed at increasing current densities demonstrated that the intensity of the dissolution–recrystallization process scales with the electrochemical driving force. Under cathodic bias, copper oxides transiently dissolve into the electrolyte, likely forming aqueous Cu(I) transient species, which are then reduced to metallic Cu and reprecipitate. In contrast, tin oxide remains chemically and structurally stable, showing no participation in this dissolution–reprecipitation cycle. These findings confirm the protective function of the SnO_2_ shell and provide direct visual evidence of its stabilizing role toward the underlying Cu_2_O phase.

Moreover, the dissolution–recrystallization phenomenon observed during operando EC‐LPTEM explains the loss of selectivity towards CO, which was experimentally observed in the 120 cm^2^ electrolyzer.

Overall, the operando EC‐LPTEM results validate the conceptual design of the Cu_2_O–SnO_2_–Re catalyst, and provide insights into how its structure influences product selectivity. Moreover, this study highlights the capability of EC‐LPTEM to resolve structural and compositional transformations of CO_2_RR catalysts at the nanoscale under varying current densities. This approach therefore represents a powerful tool for bridging the gap between lab‐scale electrochemical testing and nanoscale mechanistic understanding, enabling the rational design of more durable and efficient electrocatalysts for CO_2_ reduction.

## Supporting Information

Additional supporting information can be found online in the Supporting Information section.

## Funding

This work was supported by H2020 Leadership in Enabling and Industrial Technologies (862192) and Ministero dell’Istruzione, dell’Università e della Ricerca (NEXT GEN EU ‐ iEntrance ‐ concession decree of 21/06/2022 n. 128, PON FSE‐EU ministerial decree n. 1062/2021).

## Supporting information

Supplementary Material
