# Supplementary material for: Operando Transmission Electron Microscopy Insights Into the Evolution of Cu2O–SnO2‐Based Catalysts During CO2RR
Source: ChemSusChem. 2026 Apr 22;19(8):e202502705. doi: 10.1002/cssc.202502705 (PMC13102550; doi:10.1002/cssc.202502705)
Supplement: Supplementary file 1 — Supplementary Material [file CSSC-19-e202502705-s001.pdf]

Operando TEM Insights into the Evolution of Cu_2O-SnO_2 Based Catalysts during CO_2RR

Cecilia Irene Gho[a],[b], Katarzyna Bejtka* [a],[b], Marco Fontana[a],[b], Federica Zammillo[b], Hilmar Guzmàn[b], Micaela Castellino[a],[b], Alberto Lopera[c], Mariajose Lòpez-Tendero[c], Roger Miró[d], Miriam Díaz de los Bernardos[d], Simelys Hernàndez[b] and Angelica Chiodoni*[a]

[a] C.I. Gho, Dr. K. Bejtka, Dr. M. Fontana, Dr. Micaela Castellino, Dr. A. Chiodoni
Center for Sustainable Future Technologies @Polito
Istituto Italiano di Tecnologia
Via Livorno 60, 10144 Torino, Italy
E-mail: angelica.chiodoni@iit.it

[b] C.I. Gho, Dr. K. Bejtka, Dr. M. Fontana, Dr. F. Zammillo, Dr. H. Guzmàn, Dr. Micaela Castellino, Prof. S. Hernàndez
Department of Applied Science and Technology
Politecnico di Torino
Corso Duca degli Abruzzi 24, 10129 Torino, Italy
E-mail: katarzyna.bejtka@polito.it

[c] Dr. A. Lopera, Dr. M. Lòpez-Tendero
Laurentia Technologies
Parque Tecnologico, Avda. Benjamin Franklin 12 Paterna, Valencia 46980, Spain

[d] Dr. R. Miró, Dr. M. Díaz de los Bernardos
Unitat de Tecnologia Química
Eurecat, Centre Tecnològic de Catalunya
C/Marcel·lí Domingo 2, 43007 Tarragona, Spain

SUPPORTING INFORMATION

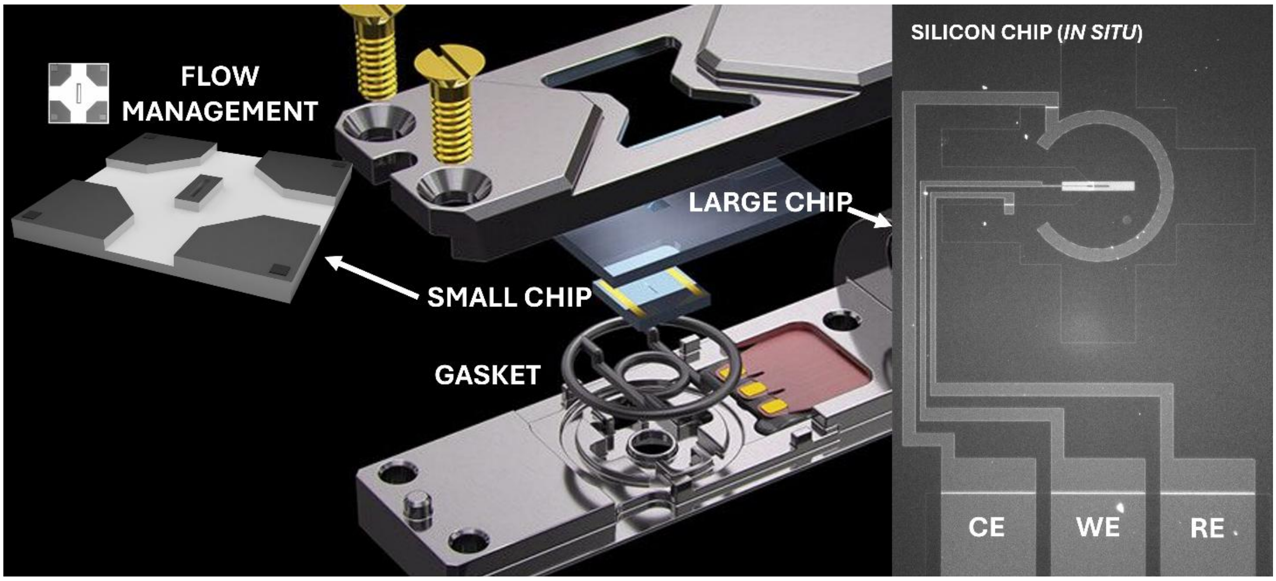

Figure S1: Exploded view of the EC-LPTEM cell with a 3D scheme of the flow management small chip (on the left) and a FESEM image of the electrochemical large chip (on the right). The different electrodes on the large chip are indicated on the respective pads.

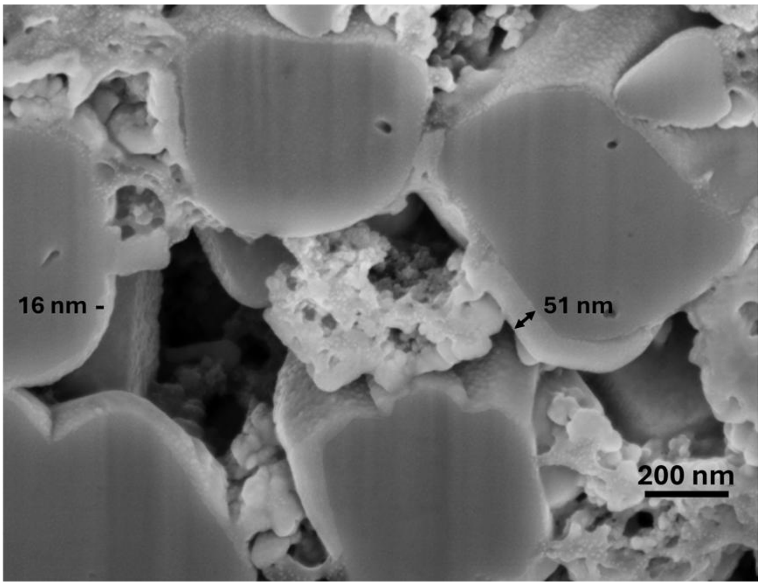

Figure S2: Cross section FESEM image of Cu_2O-SnO_2-Re catalyst. The granular SnO_2 shell is visible around the Cu_2O core, with thickness varying locally.

Supporting Note 1: XPS characterization

X-ray photoelectron spectroscopy (XPS) measurements were performed to study the surface chemical states and interfacial electronic structure of Cu-Sn catalysts on the smaller electrodes, with and without Re complex, before and after electrochemical CO_2 reduction tests. A bare metallic Cu sample was also included in the investigation as a reference to compare its signals with those of our samples. The survey spectra, not reported, highlighted the presence of Cu, Sn, F, C, and O for all samples, Si and Re for the catalyst with Re complex, and K, N, S, and Cl as solvents and electrolytes residues for the tested electrodes. The Br3d signal was not detected in the Cu_2O-SnO_2-Re catalysts (before and after the test), as its region overlapped with Cu3p.

High-resolution (HR) spectra of the Cu2p and Sn3d regions were used to investigate charge redistribution and interfacial bonding in the Cu–Sn–O system, together with Re4f region, to infer the presence of the VTES-Re complex.

Starting from the Cu2p doublet region, as reported in **Figure S3**, due to the strong overlap of the binding energies for Cu(0) and Cu(I), these oxidation states cannot be unambiguously distinguished by XPS. In contrast, the presence of shake-up satellite features (940–945 eV range) provides direct evidence for the formation of Cu(II) species. To obtain a more rigorous quantification of the Cu(II) component, according to the method reported by M. Biesinger [1], we performed the deconvolution of the $Cu2p_{3/2}$ region and its satellite, obtaining the following values for the electrodes under examination: 31% for Cu_2O-SnO_2-Re tested and 24% for the Cu_2O-SnO_2-Re pristine electrodes respectively. This result highlights the fact that the presence of the Re-containing complex reduces the surface formation of Cu(II), both before and after the catalytic test.

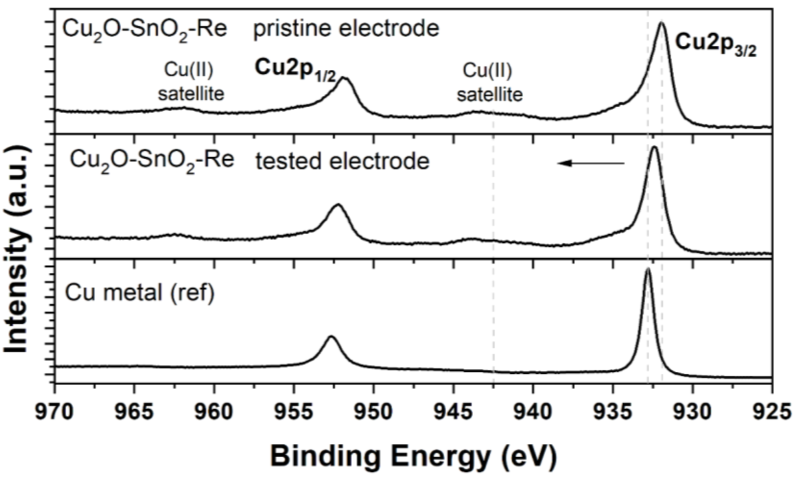

Figure S3: XPS Cu2p HR regions for samples (from top to bottom): Cu_2O-SnO_2-Re pristine electrode, Cu_2O-SnO_2-Re tested electrode and Cu metal foil as comparison.

To overcome the intrinsic limitation of the Cu2p region in discriminating between Cu(0) and Cu(I) species, the CuLMM Auger transition was measured (**Figure S4**), and the modified Auger parameter (MAP) was employed for a reliable determination of the copper chemical states [2].

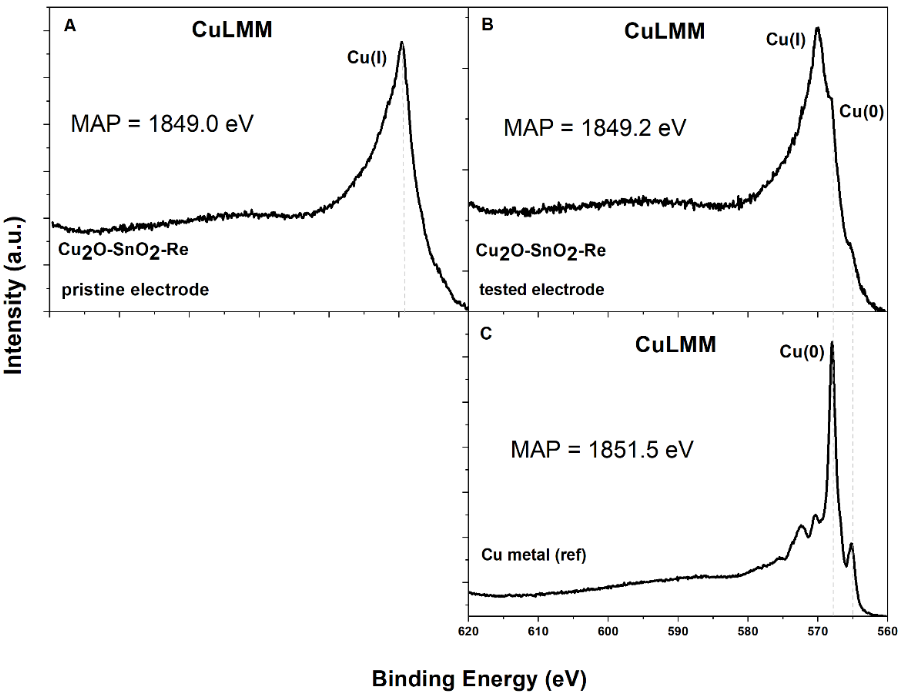

Figure S4: XPS CuLMM HR regions for samples: a) Cu_2O-SnO_2-Re pristine electrode, b) Cu_2O-SnO_2-Re tested electrode, c) Cu metal foil as comparison. MAP are also reported in each graph, for comparison.

The calculated modified Auger parameter (MAP) values in the range of 1848.9-1849.2 eV indicate that copper is predominantly present in the Cu(I) oxidation state, slightly shifted with respect to bulk Cu_2O [3], suggesting an electronically perturbed Cu environment due to strong interfacial interactions with SnO_2 (i.e. Cu-O-Sn). On the other hand, the Cu metal foil shows exactly the MAP reported in the literature, equal to 1851.5 eV. In addition to the position of the most intense peak in the CuLMM region and the values obtained for the MAP parameters, if we carefully observe the graph relating to sample Cu_2O-SnO_2-Re tested electrode (**Figure S4b**), we can notice how the Auger peak presents a first shoulder at around 568 eV and a second at around 565 eV. Comparing these two structures with the reference spectrum of metallic Cu (**Figure S4c**), we can see how they correspond exactly to the characteristic peaks of the CuLMM region of Cu(O). This implies that the electrode containing the complex with Re, after the test, shows the appearance of a metallic component for Cu, which is not visible either in the electrode before the test, nor in the tested one that does not contain the complex with Re. This result agrees with what was seen during the in-operando TEM measurement, in which the dissolution of the Cu_2O cubes not completely covered by SnO_2 was observed, and their reappearance in the form of agglomerates of metallic nanoparticles, decorating the Cu_2O- SnO_2 cubes.

The Sn presence in the catalyst was assessed, observing its behavior in the Sn3d region (**Figure S5**).

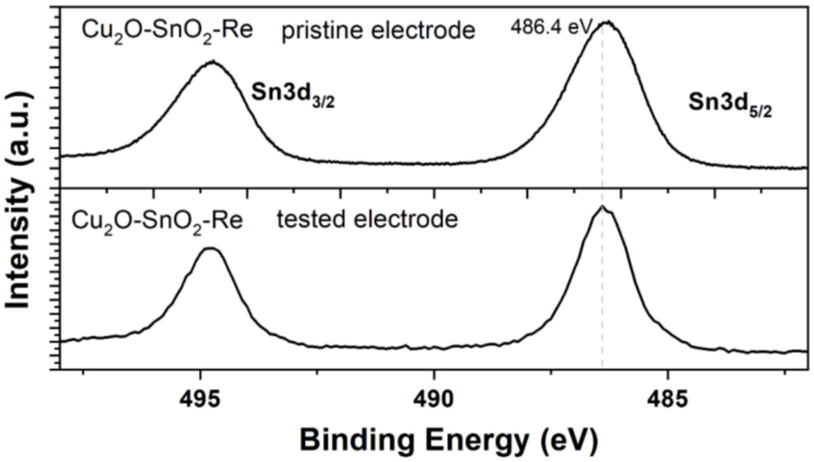

Figure S5: XPS Sn3d HR regions for samples (from top to bottom): Cu_2O-SnO_2-Re pristine electrode, b) Cu_2O-SnO_2-Re tested electrode and Cu_2O-SnO_2 without Re-complex tested electrode.

The two reported spectra first show the presence of a simple doublet which does not highlight the co-presence of different oxidation states (for example we have no significant figures in the region around 485 eV, where the metallic component of Sn should be present). The Sn3d$_{5/2}$ peaks, located in the range 485.9 – 486.4 eV, are slightly shifted to lower binding energy with respect to bulk SnO_2 [4], indicating Sn(IV) species in an electronically perturbed environment, consistent with strong interfacial interactions in the Cu–O–Sn system.

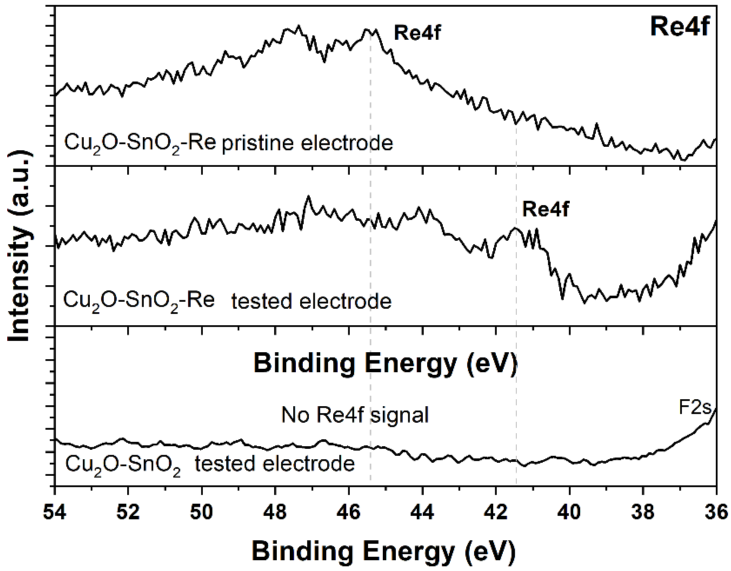

Figure S6: XPS Re4f HR regions for samples (from top to bottom): Cu_2O-SnO_2-Re pristine electrode, b) Cu_2O-SnO_2-Re tested electrode and Cu_2O-SnO_2 without Re-complex tested electrode.

Finally, we observed the Re4f region (**Figure S6**) to validate the presence of Re on the catalyst surface. For completeness, we also acquired the same region for the tested electrode that does not contain the VTES complex. Due to the low Re concentration (estimated around 0.5% at. from the HR spectra), the acquisition of the region required rather long integration times, to discern a more intense signal compared to the surrounding background.

Unfortunately, it is known that prolonged exposure of Re to X-rays can alter its oxidation state, decreasing it as the measurement progresses [5]. In the case of the pristine electrode, the measurement in this region lasted 180 minutes, while in the tested electrode it lasted 250 minutes. We can observe that (fig.4), despite the long acquisition times, the signals are very noisy. However, when compared with the region of the tested sample not containing the Re-based complex, we can observe a substantial difference. We do not want to delve into a deconvolution procedure of the found peaks, as this would be forced given the nature of the signal, we have available. We can, however, state that Re is present, in a range of values from Re(VI) to Re(IV), without knowing what our contribution was to the variation in the intrinsic oxidation state of the element. Having to choose between a short measurement, which would not have allowed us to highlight the presence of Re, and a much longer one, which however could have influenced its chemical value, we chose the latter.

Overall, the XPS analysis demonstrates the formation of a strongly coupled Cu–O–Sn interfacial system, characterized by charge redistribution and the stabilization of electronically perturbed Cu(I) and Sn(IV) species, which is likely responsible for the observed catalytic behavior.

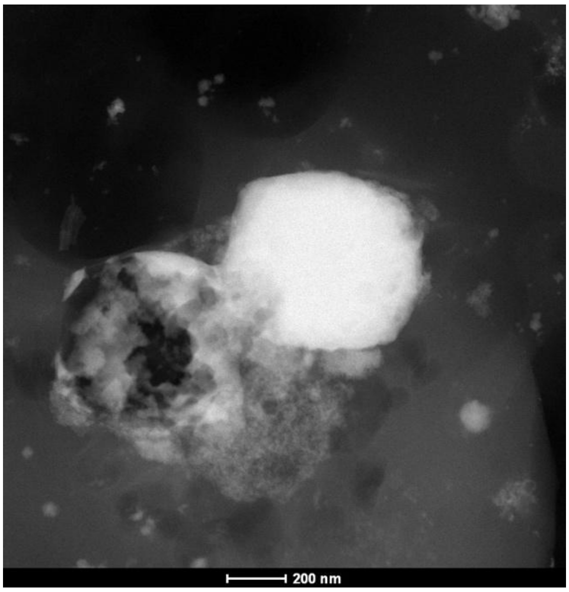

Figure S7: Image of Cu_2O-SnO_2-Re catalyst after chronopotentiometry at -2 mA/cm^2 (current applied -50 nA), showing the dissolution of cubic particles.

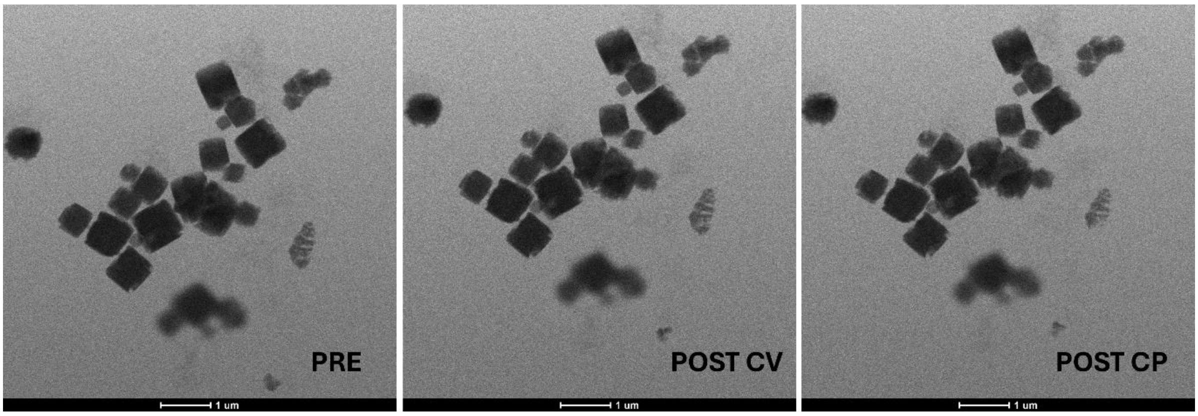

Figure S8: In situ STEM imaging of cubes on the viewing window but outside the electrode. These particles were not exposed to electrochemical stimulation, showing that the catalyst is stable in liquid environment, which do not triggers the formation of nanoparticles.

Supporting Note 2: Electrochemical measurements at 10 cm^2 scale

During the first months of the European-funded Suncochem project, an ElectroCell Micro Flow Cell® was used to assess the performance at a 10 cm^2 scale. To this end, an Ir-MMO plate was employed as the anode, and a leak-free Ag/AgCl reference electrode was inserted into the cathodic compartment. The Cu_2O/SnO_2-Re GDE was used as the working electrode (WE) with an active area of 10 cm^2. A three-compartment configuration was employed for electrochemical measurements. The WE was exposed on one side to the catholyte and on the other to the CO_2 gaseous stream. The catholyte, a 0.1 M KHCO3 aqueous solution, was circulated at 50 mL min^{-1} using a piston pump. An aqueous solution of 0.1 M KOH was used as the anolyte and circulated at 50 mL min^{-1} using a peristaltic pump (Kamoer Fluid Tech (Shanghai) Co., Ltd.). Both streams required two external reservoirs, each containing 50 mL. The CO_2 flow rate was set to 20 mL min^{-1}, using a mass flow controller (EL-FLOW Select, Bronkhorst). The electrochemical cell was operated under ambient conditions, with an overpressure of 15 mbar at the outlet gas stream.

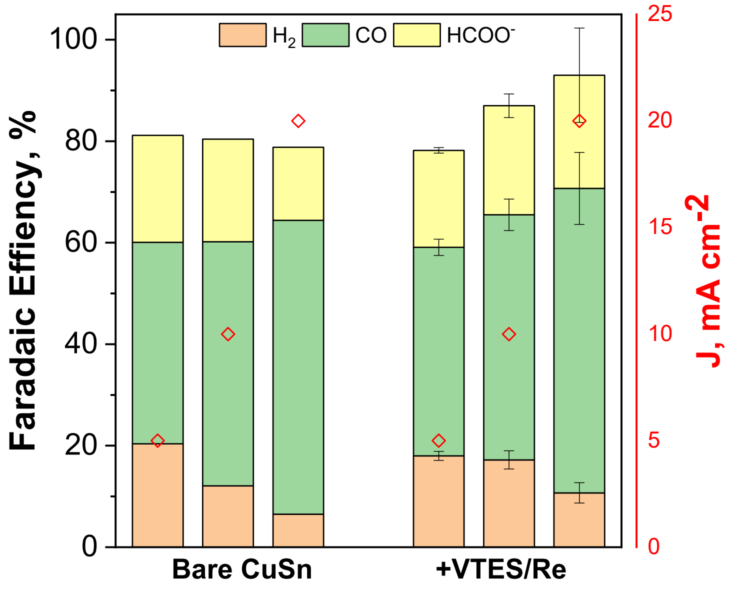

Figure S9: Effect of Re complex on the CO_2R performance of Cu_2O/SnO_2-based GDE in 0.1M $KHCO_3$ aqueous electrolyte at 10 cm^2 scale. The electrochemical tests were carried out over a current density range of 5-20 mA cm^{-2} (red symbols refer to right y-axis). Error bars represent the standard deviation calculated from two independent experiments. For Bare CuSn, only one measurement is available; therefore, error bars cannot be reported. Based on the reproducibility observed for the other sample (+VTES/Re) under identical conditions, comparable variability is expected, although it has not been quantified.

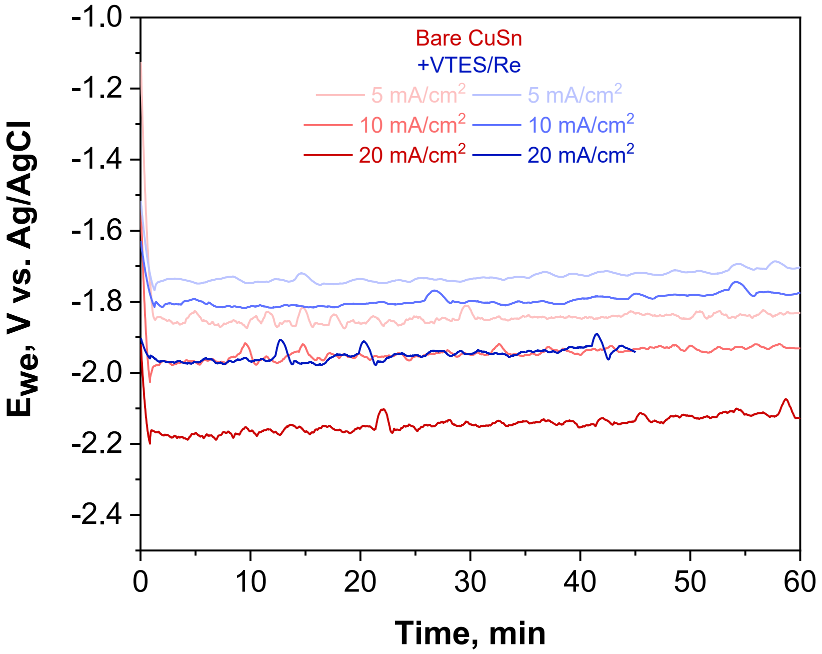

Figure S10: Impact of Re-complex on the measured potential during chronopotentiometry (5-20 mA cm^{-2}) on a Cu_2O/SnO_2-based GDE in 0.1M $KHCO_3$ aqueous electrolyte at a 10 cm² scale. The color gradient shows the rise in current density.

Table S1: Average values of measured working potential across independent tests with or without the Re complex as a co-catalyst.

		Avg. Ewe (V vs. Ag/AgCl) measured @		
**Test #**	**Catalyst**	**5 mA cm^{-2}**	**10 mA cm^{-2}**	**20 mA cm^{-2}**
1	+VTES-Re	-1.76	-1.87	-2.01
2	+VTES-Re	-1.73	-1.79	-1.95
3	Bare	-1.83	-1.98	-2.28

Table S2: Faradaic efficiencies (FE) for CO, H_2, and formate measured using a 120 cm² Cu_2O–SnO_2–Re gas diffusion electrode (GDE) in 0.1 M $KHCO_3$ at constant current densities of −6 and −20 mA cm^{-2}. Values are given as mean ± standard deviation (SD) from *n* = 3 independent replicate runs.

J, mA/cm^2	FE H_2, %	±STD	FE CO, %	±STD	FE Formate, %	±STD
**-6**	9.82	2.16	85.66	1.16	-	
**-20**	31.05	3.15	30.13	4.82	27.69	6.78

Supporting Note 3: Feed composition effect on CO_2R performance at 120 cm^2 scale

To evaluate whether CO_2 availability contributes to the observed decrease in CO selectivity at high current density, we performed a screening experiment in which the inlet gas composition was switched from 70% CO_2 (balanced with N_2) to 100% CO_2 while maintaining constant gas and electrolyte flow rates (gas feed: 90 N mL min^{-1}; electrolyte: 60 mL min^{-1}). The response of selectivity was quantified by the average CO/H_2 ratio obtained from product analysis at two current densities (−6 and −20 mA cm^{-2}). At −6 mA cm^{-2}, increasing the inlet CO_2 fraction leads to an increase in the CO/H_2 ratio (Figure S4a), from 2.8 to 3.4, consistent with improved CO_2 availability. In contrast, at −20 mA cm^{-2} the CO/H_2 ratio decreases markedly, from 3.4 to 1.8, even under 100% CO_2 feed (Figure S11a), indicating that high-rate operation introduces stronger transport and/or local-environment constraints. The gas outlet composition measured by in-line microGC (CO_2 and N_2 profiles, Figure S11b) provides a quantitative device-level indication of CO_2 conversion during the test and supports that CO_2 utilization increases during operation. Overall, these data support the interpretation that limited effective CO_2 availability can contribute to reduced CO selectivity at higher current density under the tested operating conditions, while not excluding additional effects such as catalyst reconstruction and local pH changes.

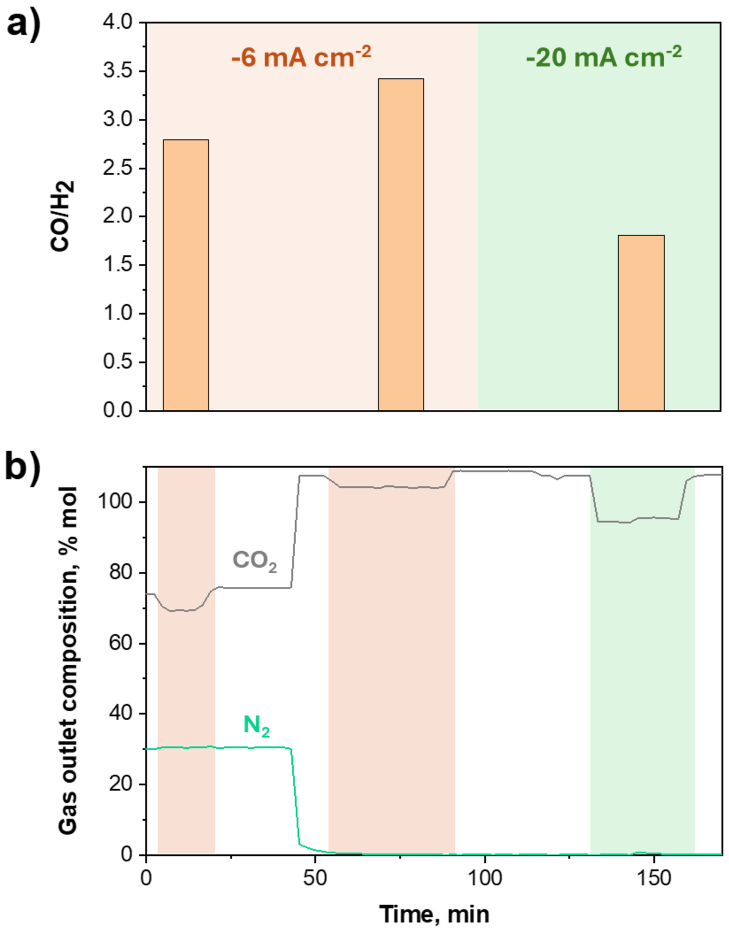

Figure S11: Correlation between electrochemical operating conditions and gas composition: (a) CO to H_2 ratio trend at different current densities (highlighted by colored bands) during CO_2R on a Cu_2O/SnO_2-Re GDE in 0.1M $KHCO_3$ aqueous electrolyte at 120 cm^2 scale; (b) gas outlet composition analyzed through inline micro gas chromatography corresponding to the same operating conditions shown in the top panel. The reduction in CO_2 concentration within the colored bands is associated with its conversion during CO2RR.

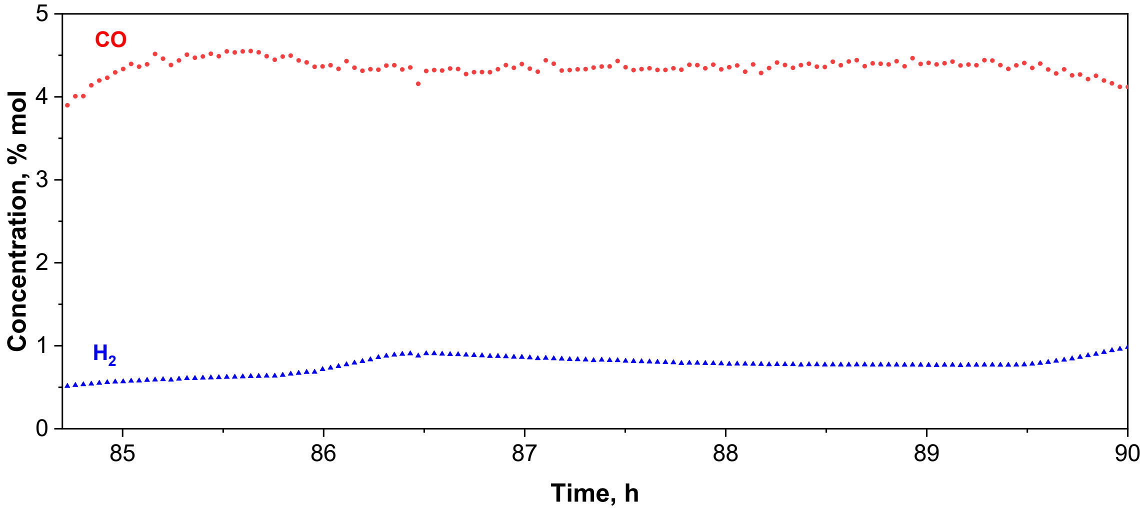

Figure S12: Gas product trends during a 5-hour chronopotentiometry at -6 mA cm^{-2} showing stable activity and selectivity during CO_2R reaction on a Cu_2O/SnO_2-Re GDE in 0.1M $KHCO_3$ aqueous electrolyte at 120 cm^2 scale.

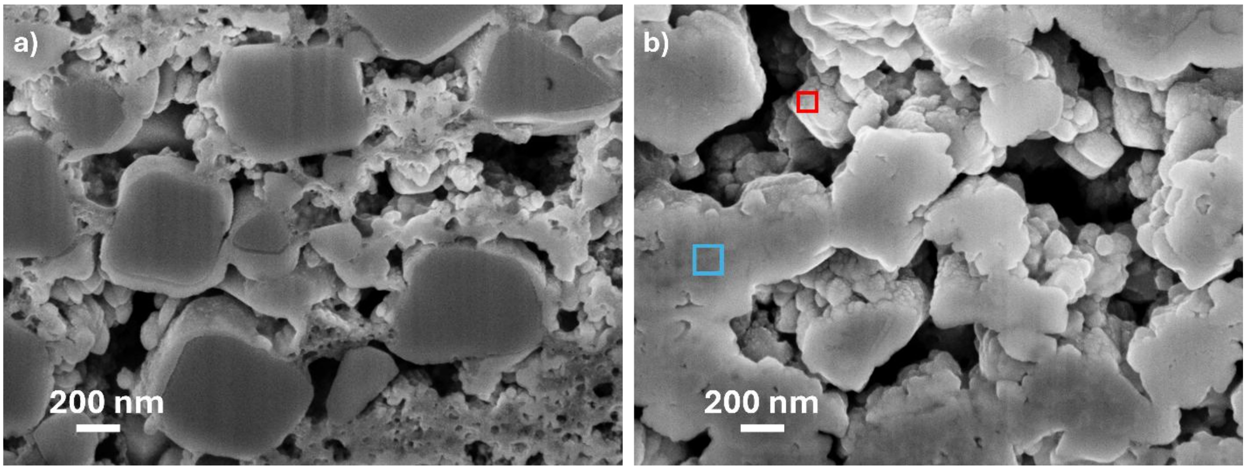

Figure S13: Cross-section view of (a) pristine and (b) tested electrodes.

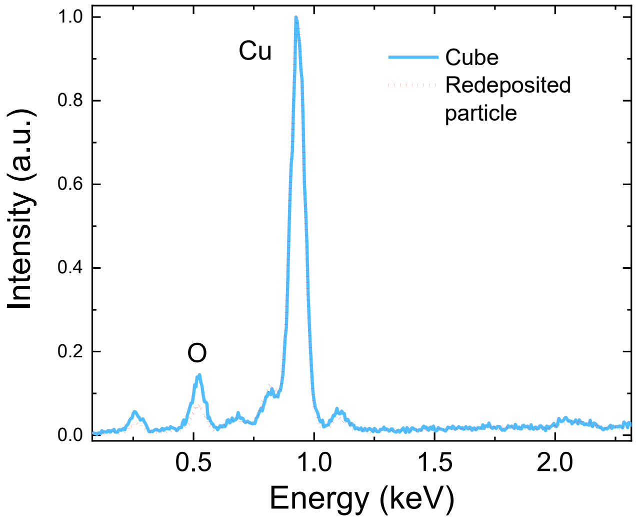

Figure S14. EDS spectra showing the chemical composition of the different types of particles present in the tested electrode. The relative position of the EDS acquisition is marked with the corresponding color in Figure S9 (b).

Table S3: EDS semi-quantitative analysis of the as-prepared and tested lab-scale electrode in FESEM.

	**Atomic %**		
	**Pristine electrode**	**Tested electrode**	
		Average	Newly formed particles
**C**	17.6	30.7	20.5
**O**	28.3	11.8	11.8
**F**	13.8	21.1	8.3
**Al**	0.2	0.1	0.0
**Si**	0.1	0.1	0.0
**Cl+K**	3.5	1.2	0.0
**Cu**	34.9	34.4	58.2
**Sn**	1.6	0.6	1.1

References

[1] M. C. Biesinger, “Advanced analysis of copper X-ray photoelectron spectra,” *Surface and Interface Analysis*, vol. 49, no. 13, pp. 1325–1334, Dec. 2017, doi: 10.1002/sia.6239.

[2] M. C. Biesinger, L. W. M. Lau, A. R. Gerson, and R. St. C. Smart, “Resolving surface chemical states in XPS analysis of first row transition metals, oxides and hydroxides: Sc, Ti, V, Cu and Zn,” *Appl. Surf. Sci.*, vol. 257, no. 3, pp. 887–898, Nov. 2010, doi: 10.1016/j.apsusc.2010.07.086.

[3] J. F. . Moulder and Jill. Chastain, *Handbook of x-ray photoelectron spectroscopy : a reference book of standard spectra for identification and interpretation of XPS data*. Physical Electronics Division, Perkin-Elmer Corp., 1992.

[4] C. D. Wagner, A. V. Naumkin, A. Kraut-Vass, J. W. Allison, C. J. Powell, and J. R. Jr. Rumble, “NIST Standard Reference Database 20, Version 3.4,” http:/srdata.nist.gov/xps/.

[5] S. Iqbal, M. L. Shozi, and D. J. Morgan, “X-ray induced reduction of rhenium salts and supported oxide catalysts,” *Surface and Interface Analysis*, vol. 49, no. 3, pp. 223–226, Mar. 2017, doi: 10.1002/sia.6076.
